# Association between the triglyceride–glucose index and the risk of mortality among patients with chronic heart failure: results from a retrospective cohort study in China

**DOI:** 10.1186/s12933-023-01895-4

**Published:** 2023-07-07

**Authors:** You Zhou, Chi Wang, Hebin Che, Liting Cheng, Di Zhu, Chongyou Rao, Qin Zhong, Zongren Li, Xiao Wang, Zisheng Wu, Kunlun He

**Affiliations:** 1 School of Medicine, Nankai University, No.94 Weijin Road, Nankai District, Tianjin, 300071 China; 2 Medical Big Data Research Center, Medical Innovation Research Department of PLA General Hospital, No.28 Fuxing Road, Haidian District, Beijing, 100853 China; 3 Chinese PLA General Hospital and Medical School, No.28 Fuxing Road, Haidian District, Beijing, 100853 China

**Keywords:** Triglyceride–glucose index, Chronic heart failure, Mortality, Metabolic syndrome, Heart failure with preserved ejection fraction

## Abstract

**Background:**

The triglyceride–glucose (TyG) index has been demonstrated to be a reliable surrogate marker of insulin resistance (IR) and an effective predictive index of cardiovascular (CV) disease risk. However, its long-term prognostic value in patients with chronic heart failure (CHF) remains uncertain.

**Methods:**

A total of 6697 consecutive patients with CHF were enrolled in this study. Patients were divided into tertiles according to their TyG index. The incidence of primary outcomes, including all-cause death and CV death, was recorded. The TyG index was calculated as ln [fasting triglycerides (mg/dL) × fasting blood glucose (mg/dL)/2].

**Results:**

During a median follow-up of 3.9 years, a total of 2158 (32.2%) all-cause deaths and 1305 (19.5%) CV deaths were documented. The incidence of primary events from the lowest to the highest TyG index tertiles were 50.61, 64.64, and 92.25 per 1000 person-years for all-cause death and 29.05, 39.40, and 57.21 per 1000 person-years for CV death. The multivariate Cox hazards regression analysis revealed hazard ratios for all-cause and CV deaths of 1.84 (95% CI 1.61–2.10; *P* for trend < 0.001) and 1.94 (95% CI 1.63–2.30; *P* for trend < 0.001) when the highest and lowest TyG index tertiles were compared. In addition, the predictive ability of the TyG index against all-cause death was more prominent among patients with metabolic syndrome and those with heart failure with preserved ejection fraction phenotype (both *P* for interaction < 0.05).

Furthermore, adding the TyG index to the established model for all-cause death improved the C‑statistic value (0.710 for the established model vs. 0.723 for the established model + TyG index, *P* < 0.01), the integrated discrimination improvement value (0.011, *P* < 0.01), the net reclassification improvement value (0.273, *P* < 0.01), and the clinical net benefit (probability range, 0.07–0.36).

**Conclusions:**

The TyG index was significantly associated with the risk of mortality, suggesting that it may be a reliable and valuable predictor for risk stratification and an effective prognostic indicator in patients with CHF.

**Supplementary Information:**

The online version contains supplementary material available at 10.1186/s12933-023-01895-4.

## Introduction

Heart failure (HF) is one of the leading causes of death and morbidity. It is estimated that 64.3 million people worldwide have HF, and the absolute number of chronic HF (CHF) patients continues to rise [1]. Furthermore, the increase in HF risk factors and the younger age of the population with HF have led this disease to place a heavy burden on human health and socio-economic development. Therefore, early identification of CHF with high residual risks for better management of the clinical risks is vital for patients.

Metabolic disorders are very prevalent in patients with HF and are associated with multiple molecular, cellular, and neurohormonal responses that may influence the prognosis of HF [2]. Insulin resistance (IR) is an important component of metabolic syndrome (MetS) that is associated with a poor prognosis in HF [3]. The hyperinsulinemic–euglycemic clamping approach is the gold standard for the diagnosis of IR and can quantify β-cell sensitivity to glucose and tissue sensitivity to insulin [4]. However, due to the time-consuming nature, high cost, and complexity of this technology, it is difficult to apply it in practical clinical settings and large-scale studies [5]. In this context, a variety of potential surrogate markers of IR have been studied and validated. Among these indicators, the Homeostasis Model Assessment of Insulin Resistance (HOMA-IR) is the most commonly used one; its values are calculated using fasting insulin and blood glucose levels, but fluctuations in insulin secretion and factors like stress or exercise may affect the accuracy of the results [5].

In recent years, many studies showed that the triglyceride–glucose (TyG) index is strongly correlated with IR, and this relationship has been previously confirmed by hyperinsulinemic–euglycemic clamping experiments [6]. Some studies have shown that the TyG index is superior to HOMA-IR in evaluating IR and predicting MetS [7]. The TyG index is based on fasting blood glucose (FBG) and triglyceride (TG) measurements, which are clinically routine and familiar, and it has been considered a reliable, simple, and economic surrogate marker of IR [8]. Several studies have shown that the TyG index is positively correlated with myocardial fibrosis, atherosclerosis, and coronary artery calcification [9, 10]. The TyG index is also related to a poor prognosis in healthy people and patients with cardiovascular (CV) diseases [11].

According to the latest clinical guidelines, HF can be divided into three phenotypes based on the measurement of left ventricular ejection fraction (LVEF) [12]. Moreover, there is evidence that different HF phenotypes are heterogeneous in their clinical manifestations and pathophysiology, which often have different effects on the prognosis and treatment of the disease [13].

To our knowledge, few studies to date have investigated the association between the TyG index and the long-term prognosis of CHF, and the answer to whether different phenotypes of HF and different metabolic status groups affect the prognostic value of the TyG index remains uncertain. Therefore, we investigated the prognostic value of the TyG index in a large sample of CHF patients and, for the first time, explored its prognostic role in different HF phenotypes and different metabolic status groups.

## Methods

**Fig. 1:**
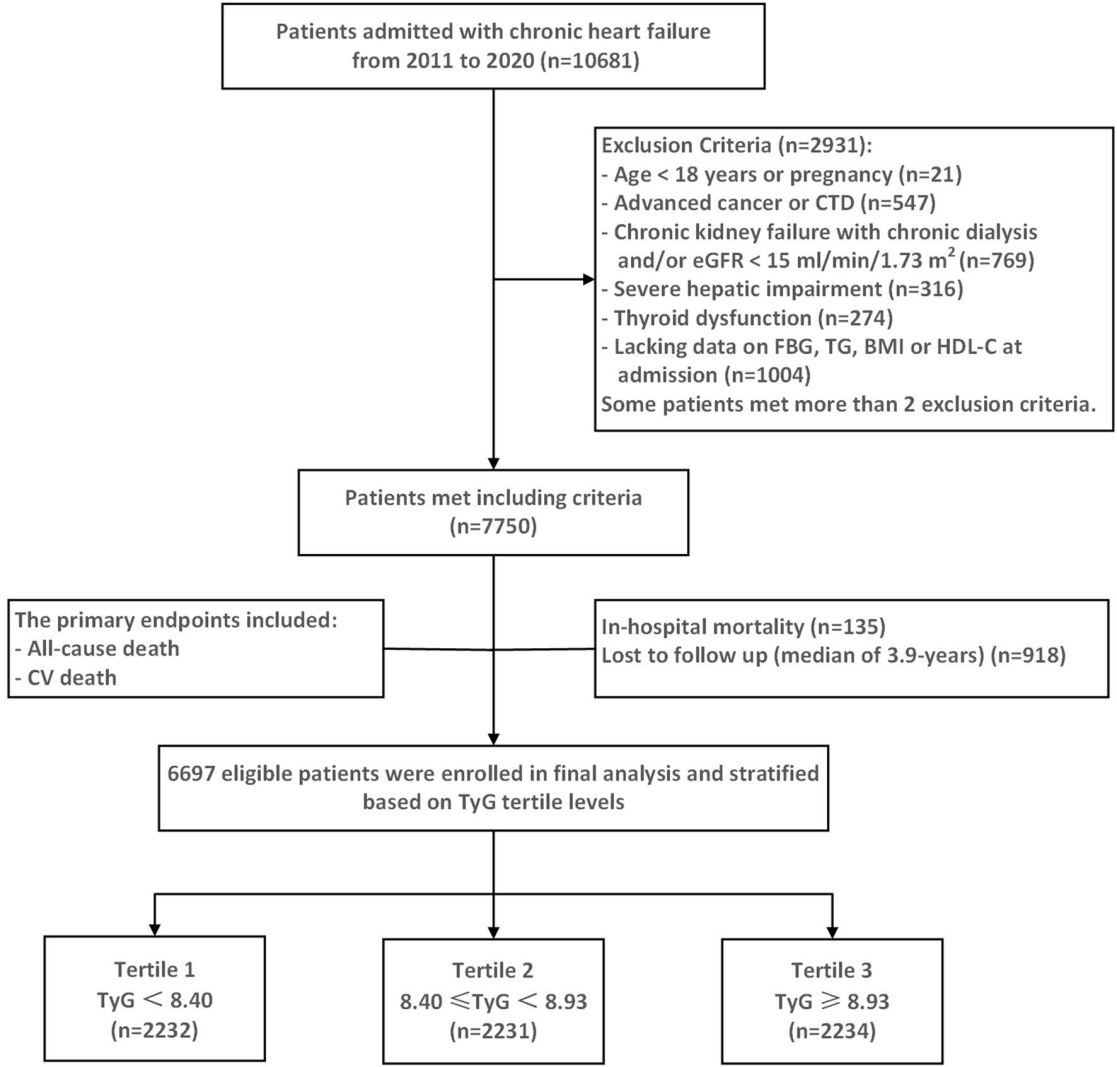
Flow diagram of patients selection. *CTD* connective tissue diseases, *eGFR* estimated glomerular filtration rate, *FBG* fasting blood glucose, *TG* triglyceride, *BMI* body mass Index, *HDL-C* high density lipoprotein cholesterol, *CV death* cardiovascular death, *TyG index* triglyceride–glucose index

In this study, a retrospective analysis was performed on 10,681 consecutive CHF patients admitted to PLA General Hospital between January 1, 2011, and December 31, 2020. CHF was defined according to the 2021 European Society for Cardiology Guidelines for the Diagnosis and Treatment of Acute and Chronic Heart Failure [12]. Among the 10,681 patients, 3984 patients were excluded based on the study exclusion criteria, which included (1) age < 18 years or pregnancy; (2) advanced cancer or connective tissue diseases; (3) chronic kidney failure with chronic dialysis and/or an estimated glomerular filtration rate (eGFR) of < 15 mL/min/1.73 m^2^; (4) severe hepatic impairment (cirrhosis with ascites or alanine aminotransferase and/or aspartate aminotransferase levels higher than five times the upper limit of normal); (5) thyroid dysfunction (hyperthyroidism or hypothyroidism), (6) lacking data on FBG, TG, body mass index (BMI), or high-density lipoprotein cholesterol (HDL-C) at admission; (7) in-hospital mortality; and (8) lost to follow up. Finally, a total of 6697 patients (4579 men and 2118 women) were enrolled in this study, with a mean age of 63.3 ± 14.2 years. In addition, all patients were divided into three groups as follows according to the tertiles of the TyG index levels: T1 (TyG index < 8.40, n = 2232), T2 (8.40 ≤ TyG < 8.93, n = 2231), and T3 (TyG index ≥ 8.93, n = 2234) (Fig. 1).

### Ethical statement

This retrospective study was conducted in accordance with the Declaration of Helsinki and approved by the ethics committee of PLA General Hospital (S2023-065-01). Because of the retrospective design of this study, the need for informed consent was waived by the institutional review board, and information related to patient identity was concealed.

### Data collection and definitions

Patient demographics, vital signs, medical history, laboratory test results, echocardiographic data, and medications were collected from the electronic medical recording system. BMI was calculated as weight divided by height squared, and the result was expressed in kg/m^2^. Smoking status included current smoker, former smoker, and never smoker. Drinking status included current drinker, former drinker, and never drinker. Fasting venous blood samples were collected for laboratory indicators. The TyG index was calculated as ln [fasting TG (mg/dL) × FBG (mg/dL)/2] [8], where a TG value of 1 mmol/L = 88.6 mg/dL and an FBG value of 1 mmol/L = 18 mg/dL. Hypertension was defined as systolic blood pressure ≥ 140 mmHg and/or diastolic blood pressure ≥ 90 mmHg and/or the use of antihypertensive drugs and/or a self-reported history of hypertension. Diabetes was defined by FBG ≥ 7.0 mmol/L and/or random blood glucose ≥ 11.1 mmol/L and/or use of hypoglycemic agents and/or a prior diagnosis of diabetes made by a physician. Chronic kidney disease was defined as an eGFR < 60 mL/min per 1.73 m^2^ (including stage III defined as an eGFR of 30–59 mL/min per 1.73 m^2^ and stage IV defined as an eGFR of 15–29 mL/min per 1.73 m^2^), and eGFR was calculated using the Chronic Kidney Disease Epidemiology Collaboration equation [14, 15]. Peripheral arterial disease was defined as intermittent claudication and/or arterial occlusive disease of the lower extremities [16]. The patient prognosis score was calculated according to the scoring scale provided by the Meta-analysis Global Group in Chronic Heart Failure (MAGGIC) study [17].

According to the 2021 ESC Guidelines for the Diagnosis and Treatment of Acute and Chronic Heart Failure [12], patients with CHF were divided into three categories: HF with reduced EF (HFrEF) (LVEF ≤ 40%), HF with mildly reduced EF (HFmrEF) (40% < LVEF < 50%), and HF with preserved EF (HFpEF) (LVEF ≥ 50%). According to the China Guidelines for Type 2 Diabetes developed by the diabetes branch of the Chinese Medical Association and the definition of obesity designated by the Working Group on Obesity in China [18] and using BMI instead of waist circumference, MetS was defined by the existence of three or more of the following abnormalities [19]: (1) obesity (BMI ≥ 28 kg/m^2^); (2) hyperglycemia (FBG ≥ 6.1 mmol/L or oral glucose tolerance test 2 h plasma glucose ≥ 7.8 mmol/L and/or confirmed diabetes under treatment); (3) elevated blood pressure (blood pressure ≥ 130/85 mmHg and/or diagnosed hypertension and on antihypertensive therapy); (4) fasting TG ≥ 1.7 mmol/L; and (5) fasting HDL-C < 1.04 mmol/L.

### Follow-up and outcomes

The median follow-up duration was 3.9 (interquartile range, 2.8–6.6) years. Prognostic information was obtained by trained physicians through telephone interviews with patients or their families or by reviewing relevant medical records. The primary outcomes in the current study included all-cause death and CV death; the latter mainly referred to death due to HF, malignant arrhythmia, myocardial infarction, sudden death, or another cardiac cause.

### Statistical analysis

The characteristics of the participants were described according to the tertiles of the TyG index. The normal distribution was verified by the Kolmogorov–Smirnov test. Continuous variables were expressed as mean ± standard deviation values or median with interquartile range values according to the presence or absence of normal distribution. Continuous data were compared using one-way analysis of variance (normal distribution) or the Kruskal–Wallis test (skewed distribution). Categorical variables were expressed as numbers and percentages, and comparisons between groups were performed using the chi-squared test or Fisher’s exact test. Propensity score matching was used to adjust for the primary confounding covariates to ensure comparability across groups in the analysis of baseline characteristics.

The cumulative incidence of the primary endpoints was described by the Kaplan–Meier method and compared between groups using the log-rank test. Univariate and multivariate Cox proportional hazards regression analyses were used to evaluate the relationship between the TyG index and the occurrence of the primary endpoints. Risk factors that were statistically significant in the univariate analysis (*P* < 0.05) and/or clinically significant were selected as covariates in the multivariate Cox model. In addition, collinearity and correlation between variables were considered in the multivariate analysis. The TyG index was examined as a categorical variable (using the lowest tertile as the reference) or continuous variable (per one unit increment), and the results were expressed with hazard ratio (HR) and 95% confidence interval (CI) values. Besides the unadjusted model, two other models were fitted, including model 1, which controlled for age, sex, BMI, smoking status, drinking status, hemoglobin, alanine transaminase, aspartate aminotransferase, total bilirubin, albumin, eGFR, total cholesterol, low-density lipoprotein, HDL-C, cardiac troponin T, sodium, NT-proBNP, LVEF, and New York Heart Association classification, and model 2, which was adjusted for the variables included in model 1 plus hypertension, diabetes, atrial fibrillation, previous myocardial infarction, angina, stroke, chronic obstructive pulmonary disorder, previous heart surgery, antiplatelet agents, lipid-lowering drugs, angiotensin-converting enzyme inhibitor/angiotensin receptor blocker (ACEI/ARB) therapy, angiotensin receptor-neprilysin inhibitors, β‑blocker therapy, mineralocorticoid antagonists, diuretics, digoxin, and hypoglycemic therapy. The linear trends across TyG quartiles were evaluated by a median value within each tertile as a continuous variable. Missing covariates were replaced by multiple imputations with chained equations. The results from analyses that excluded participants with missing covariates were consistent. The proportional hazards assumption was evaluated by Schoenfeld residuals, and no potential violation was observed. Moreover, to illustrate the dose–response relationship (linear or non-linear) between the TyG index and the risk of primary endpoints, restricted cubic spline analysis adjusted for variables in model 2 was performed, with three default knots at the 10th, 50th, and 90th percentiles [20]. We also performed exploratory analyses among different subgroups, and the likelihood ratio test was used for the interaction between subgroups.

Then, the incremental effect of the TyG index in risk stratification was further tested by the C-statistic, net reclassification index, integrated discrimination improvement, and decision curve analysis with the traditional baseline model (the MAGGIC model) used as a reference. All statistical analyses were calculated using R software (version 4.2.1; R Foundation for Statistical Computing, Vienna, Austria). A two-tailed *P* value of < 0.05 was regarded as statistically significant.

## Results

### Baseline characteristics of patients

Overall, a total of 6697 eligible participants were included in the analysis. The baseline characteristics of the study population according to TyG index tertiles are presented in Table 1. The median follow-up time was 3.9 years. The average age of the participants was 63.3 years, and 68.37% of the participants were male. Participants with higher baseline TyG indices had a greater prevalence of comorbidities (including hypertension, diabetes, chronic kidney disease, previous myocardial infarction, angina, and MetS) and higher ratios of HFpEF patients, current smokers, and patients with a history of PCI. Patients in this group were also more prone to using antiplatelet agents, lipid-lowering drugs, ACEIs/ARBs, β-blockers, CCB, diuretics, nitrates, and hypoglycemic therapies (including insulin and oral antidiabetic agents). Moreover, they had higher BMI, systolic and/or diastolic blood pressure, white blood cell, hemoglobin, platelet, albumin, potassium, blood urea nitrogen, FBG, TG, TC, low-density lipoprotein cholesterol, and cardiac troponin T values but lower aspartate aminotransferase, total bilirubin, eGFR, HDL-C, sodium, LDH, and NT-proBNP levels (all *P* < 0.05). Meanwhile, the highest TyG index tertile had the lower proportions of patients with atrial fibrillation, cardiac valve surgery, and the use of mineralocorticoid antagonists and digoxin (all *P* < 0.05).

**Table 1:** Baseline characteristics of the study population according to TyG index tertiles

Variables	Total	Tertile of TyG index			*P* value
		T1	T2	T3	
	n = 6697	n = 2232	n = 2231	n = 2234	
TyG index	8.69 ± 0.64	< 8.40	8.40–8.93	≥ 8.93	–
Demographics					
Age (years)	64.0 (54.3–73.7)	64.3 (54.3–73.4)	64.0 (54.5–73.8)	64.0 (54.0–73.7)	0.967
Male (%)	4579 (68.37%)	1520 (68.10%)	1539 (68.98%)	1520 (68.04%)	0.750
BMI (kg/m^2^)	25.2 (22.8–27.8)	24.0 (21.6–26.6)	25.4 (23.0–27.8)	26.0 (23.7–28.6)	** < 0.001**
Medical measurements					
SBP (mmHg)	129.0 (115.0–144.0)	127.0 (113.0–141.0)	128.0 (115.0–143.0)	131.0 (117.0–146.0)	** < 0.001**
DBP (mmHg)	75.0 (66.0–84.0)	73.0 (65.0–82.0)	75.0 (67.0–84.0)	76.0 (67.0–85.0)	** < 0.001**
HR (bpm)	79.0 (70.0–91.0)	78.0 (69.0–90.0)	78.0 (70.0–91.0)	80.0 (70.0–91.0)	0.183
Smoking (%)					**0.017**
Current smoker	1623 (24.23%)	494 (22.13%)	553 (24.79%)	576 (25.78%)	
Former smoker	1336 (19.95%)	456 (20.43%)	419 (18.78%)	461 (20.64%)	
Never smoker	3738 (55.82%)	1282 (57.44%)	1259 (56.43%)	1197 (53.58%)	
Drinking (%)					0.296
Current drinker	1549 (23.13%)	515 (23.07%)	506 (22.68%)	528 (23.63%)	
Former drinker	664 (9.91%)	218 (9.77%)	204 (9.14%)	242 (10.83%)	
Never drinker	4484 (66.96%)	1499 (67.16%)	1521 (68.18%)	1464 (65.53%)	
LVEF (%)					** < 0.001**
≤ 40%	2221 (33.16%)	831 (37.23%)	757 (33.93%)	633 (28.33%)	
41–49%	1433 (21.40%)	456 (20.43%)	493 (22.10%)	484 (21.67%)	
≥ 50%	3043 (45.44%)	945 (42.34%)	981 (43.97%)	1117 (50.00%)	
NYHA classification (%)					0.547
I-II	2892 (43.18%)	938 (42.03%)	980 (43.93%)	974 (43.60%)	
III	2939 (43.89%)	990 (44.35%)	963 (43.16%)	986 (44.14%)	
IV	866 (12.93%)	304 (13.62%)	288 (12.91%)	274 (12.26%)	
Medical history (%)					
Hypertension	4142 (61.85%)	1166 (52.24%)	1407 (63.07%)	1569 (70.23%)	** < 0.001**
Diabetes	2987 (44.60%)	492 (22.04%)	937 (42.00%)	1558 (69.74%)	** < 0.001**
AF	2134 (31.87%)	806 (36.11%)	721 (32.32%)	607 (27.17%)	** < 0.001**
CKD (Stages III-IV)	1762 (26.31%)	516 (23.12%)	555 (24.88%)	691 (30.93%)	** < 0.001**
Previous MI	2260 (33.75%)	639 (28.63%)	780 (34.96%)	841 (37.65%)	** < 0.001**
Angina	1975 (29.49%)	555 (24.87%)	678 (30.39%)	742 (33.21%)	** < 0.001**
Stroke	1275 (19.04%)	396 (17.74%)	427 (19.14%)	452 (20.23%)	0.105
PAD	993 (14.83%)	333 (14.92%)	309 (13.85%)	351 (15.71%)	0.214
COPD	266 (3.97%)	83 (3.72%)	94 (4.21%)	89 (3.98%)	0.699
MetS	3267 (48.78%)	400 (17.92%)	986 (44.20%)	1881 (84.20%)	** < 0.001**
Previous heart surgery (%)					
PCI	1296 (19.35%)	375 (16.80%)	461 (20.66%)	460 (20.59%)	** < 0.001**
CABG	269 (4.02%)	91 (4.08%)	91 (4.08%)	87 (3.89%)	0.937
Cardiac valve surgery	206 (3.08%)	85 (3.81%)	77 (3.45%)	44 (1.97%)	** < 0.001**
Pacemaker therapy	268 (4.00%)	88 (3.94%)	84 (3.77%)	96 (4.30%)	0.653
Laboratory measurements					
WBC (10^9^/L)	6.58 (5.36–8.03)	5.98 (4.89–7.23)	6.64 (5.41–8.00)	7.13 (5.95–8.66)	** < 0.001**
Hemoglobin (g/L)	135.0 (121.0–148.0)	134.0 (119.0–146.0)	136.0 (122.0–148.0)	136.0 (120.0–150.0)	** < 0.001**
Platelets (10^9^/L)	192.0 (154.0–234.0)	182.0 (145.0–223.0)	191.0 (156.0–235.5)	202.0 (164.0–244.0)	** < 0.001**
ALT (U/L)	19.4 (13.7–30.0)	19.6 (14.3–29.7)	19.3 (13.4–30.3)	19.5 (13.2–29.7)	0.149
AST (U/L)	19.5 (15.4–26.6)	19.9 (15.9–27.5)	19.6 (15.4–26.7)	18.9 (14.9–25.3)	** < 0.001**
TBil (umol/L)	12.8 (9.0–18.4)	14.1 (9.9–20.4)	12.8 (9.2–17.9)	11.5 (8.1–16.5)	** < 0.001**
Albumin (g/L)	40.0 (37.2–42.6)	39.4 (36.7–42.0)	40.2 (37.4–42.8)	40.3 (37.5–43.0)	** < 0.001**
BUN (mmol/L)	6.60 (5.19–8.58)	6.52 (5.13–8.36)	6.50 (5.12–8.40)	6.79 (5.36–9.02)	** < 0.001**
Creatinine (umol/L)	84.7 (71.3–104.5)	83.3 (70.4–100.6)	83.9 (71.1–103.8)	87.5 (72.7–111.4)	** < 0.001**
eGFR (ml/min/1.73m^2^)	77.9 (58.8–92.2)	79.8 (61.8–92.9)	77.9 (60.1–92.4)	74.9 (53.8–91.3)	** < 0.001**
FBG (mmol/L)	5.44 (4.75–7.00)	4.77 (4.37–5.27)	5.47 (4.84–6.56)	7.21 (5.73–10.07)	** < 0.001**
TG (mmol/L)	1.20 (0.87–1.71)	0.81 (0.67–0.97)	1.29 (1.07–1.51)	1.87 (1.46–2.48)	** < 0.001**
TC (mmol/L)	3.77 (3.11–4.54)	3.55 (2.96–4.22)	3.79 (3.13–4.51)	4.02 (3.29–4.91)	** < 0.001**
LDL-C (mmol/L)	2.30 (1.75–2.94)	2.14 (1.65–2.71)	2.34 (1.81–2.97)	2.44 (1.83–3.15)	** < 0.001**
HDL-C (mmol/L)	1.00 (0.82–1.19)	1.11 (0.93–1.33)	1.01 (0.84–1.18)	0.89 (0.75–1.03)	** < 0.001**
Potassium (mmol/L)	3.97 (3.68–4.28)	3.97 (3.68–4.29)	3.95 (3.67–4.25)	3.98 (3.69–4.31)	**0.039**
Sodium (mmol/L)	140.9 (138.6–142.8)	141.1 (138.7–143.0)	141.2 (138.9–143.0)	140.4 (138.1–142.4)	** < 0.001**
LDH (U/L)	186.7 (157.5–229.6)	189.9 (159.6–232.8)	186.1 (156.9–228.2)	183.9 (156.2–229.4)	**0.013**
cTnT (ng/ml)	0.024 (0.013–0.060)	0.024 (0.013–0.070)	0.023 (0.013–0.054)	0.026 (0.014–0.058)	**0.014**
NT-proBNP (pg/ml)	1621.0 (686.8–4055.0)	1858.5 (759.2–4352.2)	1536.0 (678.2–3831.0)	1524.0 (629.9–4010.2)	** < 0.001**
Medications at discharge (%)					
Antiplatelet agents	3806 (56.83%)	1078 (48.30%)	1325 (59.39%)	1403 (62.80%)	** < 0.001**
Statins	3909 (58.37%)	1094 (49.01%)	1343 (60.20%)	1472 (65.89%)	** < 0.001**
Fenofibrate	22 (0.33%)	1 (0.04%)	3 (0.13%)	18 (0.81%)	** < 0.001**
Other lipid-lowering drugs	468 (6.99%)	114 (5.11%)	151 (6.77%)	203 (9.09%)	** < 0.001**
ACEI/ARB	2873 (42.90%)	873 (39.11%)	981 (43.97%)	1019 (45.61%)	** < 0.001**
ARNI	79 (1.18%)	31 (1.39%)	20 (0.90%)	28 (1.25%)	0.290
β-blocker	4692 (70.06%)	1447 (64.83%)	1600 (71.72%)	1645 (73.63%)	** < 0.001**
CCB	1495 (22.32%)	378 (16.94%)	516 (23.13%)	601 (26.90%)	** < 0.001**
Mineralocorticoid antagonists	4366 (65.19%)	1521 (68.15%)	1448 (64.90%)	1397 (62.53%)	** < 0.001**
Diuretics	3230 (48.23%)	1031 (46.19%)	1060 (47.51%)	1139 (50.98%)	**0.004**
Nitrates	2808 (41.93%)	802 (35.93%)	965 (43.25%)	1041 (46.60%)	** < 0.001**
Digoxin	2421 (36.15%)	893 (40.01%)	806 (36.13%)	722 (32.32%)	** < 0.001**
Insulin	865 (12.92%)	141 (6.32%)	232 (10.40%)	492 (22.02%)	** < 0.001**
SGLT2 inhibitors	55 (0.82%)	18 (0.81%)	16 (0.72%)	21 (0.94%)	0.708
Other oral antidiabetic agents	1419 (21.19%)	235 (10.53%)	412 (18.47%)	772 (34.56%)	** < 0.001**
Follow-up time (years)	3.9 (2.8–6.6)	4.3 (3.1–6.8)	4.1 (3.0–6.6)	3.7 (2.4–6.1)	** < 0.001**
All-cause death					
Incident cases	2158 (32.22%)	561 (25.13%)	694 (31.11%)	903 (40.42%)	** < 0.001**
Incidence/1000 person-years	68.27	50.61	64.64	92.25	** < 0.001**
CV death					
Incident cases	1305 (19.49%)	322 (14.43%)	423 (18.96%)	560 (25.07%)	** < 0.001**
Incidence/1000 person-years	41.29	29.05	39.40	57.21	** < 0.001**

*TyG index* triglyceride-glucose index, *BMI* body mass index, *SBP* systolic blood pressure, *DBP* diastolic blood pressure, *HR* heart rate, *LVEF* left ventricular ejection fraction, *NYHA* New York Heart Association, *AF* atrial fibrillation, *CKD* chronic kidney disease, *MI* myocardial infarction, *PAD* peripheral arterial disease, *COPD* chronic obstructive pulmonary disease, *MetS* metabolic syndrome, *PCI* percutaneous coronary intervention, *CABG* coronary artery bypass grafting, *WBC* white blood cell, *ALT* alanine aminotransferase, *AST* aspartate aminotransferase, *TBil* total bilirubin, *BUN* blood urea nitrogen, *eGFR* estimated glomerular filtration rate, *FBG* fasting blood glucose, *TG* triglyceride, *TC* total cholesterol, *LDL-C* low-density lipoprotein cholesterol, *HDL-C* high-density lipoprotein cholesterol, *LDH* lactic dehydrogenase, *cTnT* cardiac troponin T, *NT-proBNP* N-terminal pro-brain natriuretic peptide, *ACEI/ARB* angiotensin converting enzyme inhibitor/angiotensin receptor blocker, *ARNI* angiotensin receptor-neprilysin inhibitors, *CCB* calcium channel blockers, *SGLT2* inhibitors sodium-glucose co-transporter-2 inhibitors, *CV* death cardiovascular death. *P* values < 0.05 are presented in bold

### Association between TyG index and the risk of outcomes

**Fig. 2:**
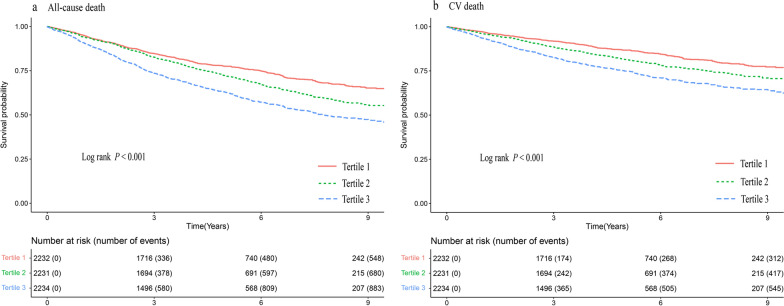
Kaplan–Meier estimation of (**a**) all-cause death and (**b**) CV death by tertiles of TyG index in HF patients. *CV death* cardiovascular death, *TyG index* triglyceride–glucose index, *HF* heart failure

During the follow-up period, all-cause death was found in 2158 (32.2%) cases and CV death occurred in 1305 (19.5%) cases. The patients who died included 561 (25.1%) in the T1 group, 694 (31.1%) in the T2 group, and 903 (40.4%) in the T3 group. The patients who died due to CV events included 322 (14.4%) in the T1 group, 423 (19.0%) in the T2 group, and 560 (25.1%) in the T3 group. The incidence of primary events from the lowest to the highest TyG index tertiles were 50.61, 64.64, and 92.25 per 1000 person-years for all-cause death and 29.05, 39.40, and 57.21 per 1000 person-years for CV death. Kaplan–Meier curves of the incidence of the primary outcomes (including all-cause death and CV death) for the TyG index tertiles are presented in Fig. 2. The results revealed that the cumulative incidence of both all-cause death and CV death increased with higher tertiles of the TyG index (log-rank test, both* P* < 0.001).

Univariate and multivariate Cox proportional hazards regression analyses are presented in Table 2. The results showed a significant association between the TyG index and all-cause death both in the unadjusted model (HR, 1.40 [95% CI 1.31–1.49]; *P* < 0.001) and fully adjusted model (HR, 1.51 [95% CI 1.38–1.64]; *P* < 0.001). Furthermore, the TyG index was also associated with CV death in both the unadjusted model (HR, 1.46 [95% CI 1.35–1.58]; *P* < 0.001) and fully adjusted model (HR, 1.54 [95% CI 1.38–1.72]; *P* < 0.001). The risk of all-cause death in the T2 and T3 TyG index groups was higher than that in the T1 TyG index group and showed a tendency to increase with the TyG index (T1 vs. T2: HR, 1.29 [95% CI 1.15–1.46]; T3: HR, 1.84 [95% CI 1.61–2.10]; *P* for trend < 0.001). Similar results were obtained in the Cox proportional risk analysis of the TyG index and CV death (T1 vs. T2: HR, 1.34 [95% CI 1.15–1.56]; T3: HR, 1.94 [95% CI 1.63–2.30]; *P* for trend < 0.001). The restricted cubic spline regression model showed a linear relationship between the TyG index and the risk of all-cause death in HF patients (*P* for non-linear association = 0.038). However, a non-linear relationship was found between the TyG index and the risk of CV death (*P* for non-linear association = 0.129) (Additional file 1: Fig. S1).

**Table 2:** HRs (95% CI) of primary outcomes according to TyG index tertiles

Categories	Events (%)	Unadjusted	*P*-value	*P* for trend	Model 1	*P*-value	*P* for trend	Model 2	*P*-value	*P* for trend
		HR (95% CI)			HR (95% CI)			HR (95% CI)		
All-cause death										
Continuous variable per 1 unit		1.40 (1.31–1.49)	** < 0.001**		1.62 (1.50–1.75)	** < 0.001**		1.51 (1.38–1.64)	** < 0.001**	
Tertile^a^	2158 (32.2)			** < 0.001**			** < 0.001**			** < 0.001**
T1 (n = 2232)	561 (25.1)	Ref			Ref			Ref		
T2 (n = 2231)	694 (31.1)	1.27 (1.14–1.42)	** < 0.001**		1.38 (1.23–1.55)	** < 0.001**		1.29 (1.15–1.46)	** < 0.001**	
T3 (n = 2234)	903 (40.4)	1.82 (1.64–2.02)	** < 0.001**		2.08 (1.84–2.35)	** < 0.001**		1.84 (1.61–2.10)	** < 0.001**	
CV death										
Continuous variable per 1 unit		1.46 (1.35–1.58)	** < 0.001**		1.66 (1.50–1.83)	** < 0.001**		1.54 (1.38–1.72)	** < 0.001**	
Tertile	1305 (19.5)			** < 0.001**			** < 0.001**			** < 0.001**
T1 (n = 2232)	322 (14.4)	Ref			Ref			Ref		
T2 (n = 2231)	423 (19.0)	1.35 (1.17–1.56)	** < 0.001**		1.45 (1.24–1.68)	** < 0.001**		1.34 (1.15–1.56)	** < 0.001**	
T3 (n = 2234)	560 (25.1)	1.96 (1.71–2.25)	** < 0.001**		2.20 (1.88–2.58)	** < 0.001**		1.94 (1.63–2.30)	** < 0.001**	

Model 1: adjusted for age, gender, body mass index, smoking status, drinking status, hemoglobin, ALT, AST, TBil, albumin, eGFR, total cholesterol, LDL-C, HDL-C, cTnT, sodium, NT-proBNP, LVEF and NYHA classification

Model 2: adjusted for Model 1 + hypertension, diabetes, atrial fibrillation, previous MI, angina, stroke, COPD, previous heart surgery, antiplatelet agents, lipid-lowering drugs, ACEI/ARB, ARNI, β-blocker, mineralocorticoid antagonists, diuretics, digoxin and hypoglycemic therapy

*CI* confidence interval, *HR* hazard ratio, *TyG index* triglyceride-glucose index

^a^TyG index: T1 (< 8.40), T2 (8.40–8.93), T3 (≥ 8.93). *P* values < 0.05 are presented in bold

Furthermore, propensity score matching analysis adjusted for the primary confounding covariates between the three groups was performed to evaluate the consistency of our results (Additional file 5: Table S1). Similar results were obtained even after adjusting for multiple factors (Additional file 5: Table S2).

### Predictive implications of the TyG index for mortality in different metabolic status groups and different HF phenotype groups

We further performed exploratory analyses with HF patients in different metabolic status groups and different phenotype groups. The Kaplan–Meier analysis showed significant differences in the risk of all-cause death among the three tertiles, both in the MetS group (log-rank test, *P* < 0.001) and the non-MetS group (log-rank test, *P* = 0.009). However, when the analysis was stratified by HF phenotype, these significant differences were only observed in the HFmrEF group and HFpEF group (log-rank test: *P* = 0.004 and *P* < 0.001, respectively) and not in the HFrEF group (log-rank test, *P* = 0.44). No matter the metabolic status or HF phenotype, similar results were obtained in the Kaplan–Meier analysis for the association between TyG index tertiles and CV death (Additional file 2: Fig. S2).

**Fig. 3:**
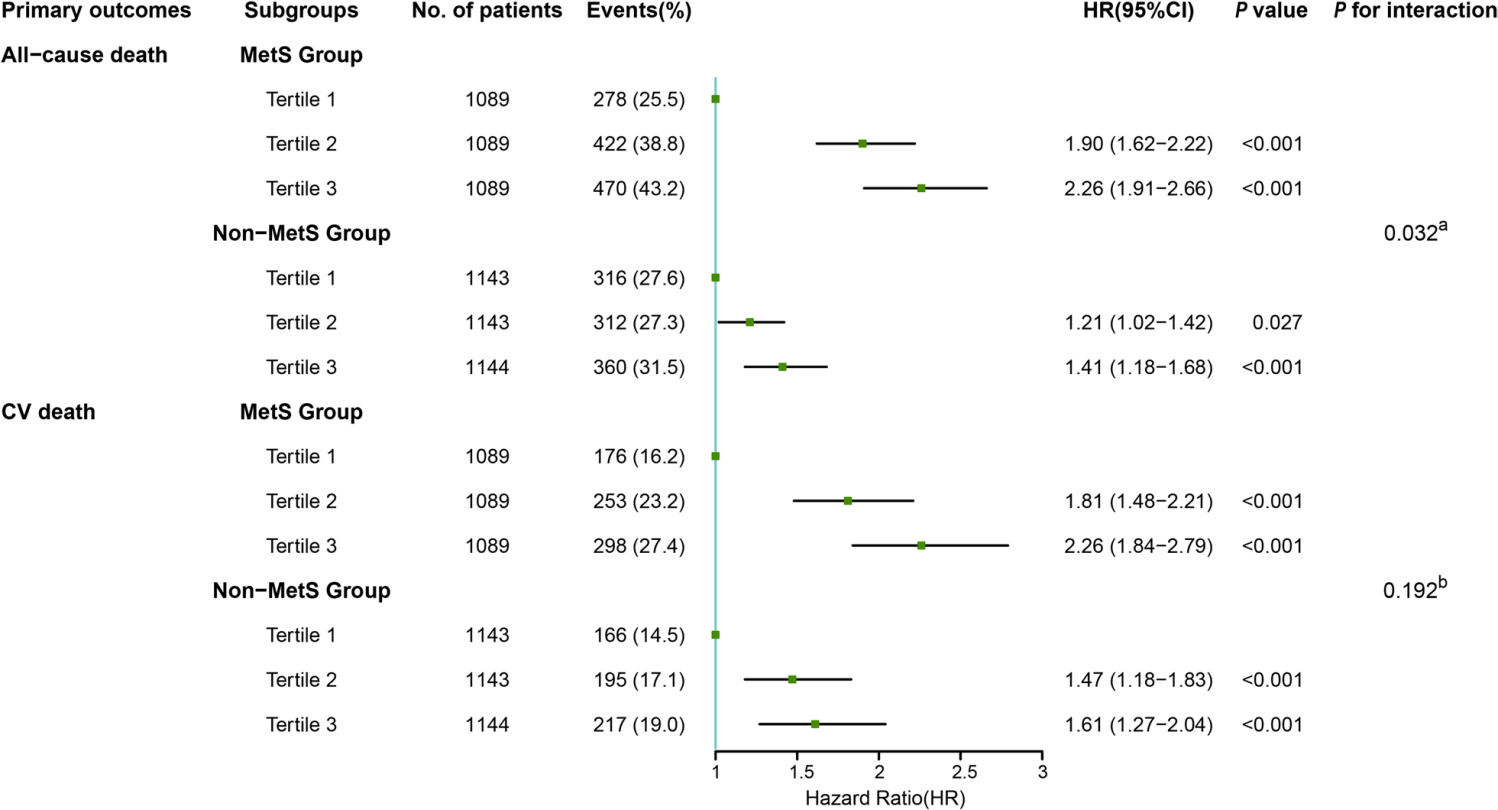
Forest plot of all-cause death and CV death according to tertiles of TyG index in HF patients with different metabolic status adjusted for model 2. *HR* hazard ratio, *CI* confidence interval, *CV death* cardiovascular death, *TyG index* triglyceride–glucose index, *HF* heart failure, *MetS* metabolic syndrome. ^a^*P* for interaction between the TyG index and the metabolic status with all-cause death as an endpoint, ^b^*P* for interaction between the TyG index and the metabolic status with CV death as an endpoint

The results of the univariate and multivariate Cox proportional hazards analyses for the association between the TyG index and primary outcomes in different metabolic status groups are presented in Additional file 5: Table S3. Following the adjustment of variates in the final model, although a higher TyG index (whether categorical or continuous) was more prone to correlating with a significant predictive potential for all-cause death in both the MetS group and non-MetS group, this phenomenon was more prominent among the MetS group (MetS group, T1 vs. T2: HR, 1.90 [95% CI 1.62–2.22]; T1 vs. T3: HR, 2.26 [95% CI 1.91–2.66] with *P* for trend < 0.001 and non-MetS group, T1 vs. T2: HR, 1.21 [95% CI 1.02–1.42]; T1 vs. T3: HR, 1.41 [95% CI 1.18–1.68] with *P* for trend < 0.001; *P* for interaction = 0.032 < 0.05). When CV death was taken as an endpoint, the results showed that the predictive value of the TyG index was similar in both groups (MetS group, T1 vs. T2: HR, 1.81 [95% CI 1.48–2.21]; T1 vs. T3: HR, 2.26 [95% CI 1.84–2.79] with *P* for trend < 0.001; and non-MetS group, T1 vs. T2: HR, 1.47 [95% CI 1.18–1.83]; T1 vs. T3: HR, 1.61 [95% CI 1.27–2.04] with P for trend < 0.001; *P* for interaction = 0.192). (Fig. 3).

**Fig. 4:**
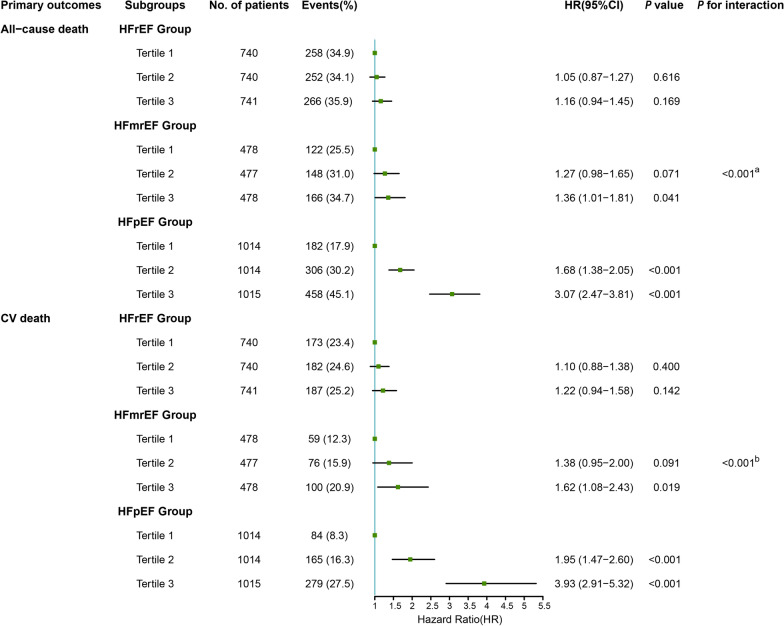
Forest plot of all-cause death and CV death according to tertiles of TyG index in different HF phenotypes adjusted for model 2. *HR* hazard ratio, *CI* confidence interval, *CV death* cardiovascular death, *TyG index* triglyceride-glucose index, *HF* heart failure, *HFrEF* heart failure with reduced ejection fraction, *HFmrEF* heart failure with mildly reduced ejection fraction, *HFpEF* heart failure with preserved ejection fraction. ^a^*P* for interaction between the TyG index and the HF phenotype with all-cause death as an endpoint, ^b^*P* for interaction between the TyG index and the HF phenotype with CV death as an endpoint

Additional file 5: Table S4 shows the association between the TyG index and primary outcomes in different HF phenotypes. When the analysis was stratified by HF phenotype, we observed a significant trend of the TyG index related to the risk of all-cause death among the non-HFrEF group (HFmrEF and HFpEF, both *P* for trend < 0.05), while no significant differences were observed in the HFrEF group (*P* for trend = 0.170). When taking CV death as an endpoint, the predictive implication of the TyG index was still obvious in the HFpEF group (T1 vs. T2: HR, 1.95 [95% CI 1.47–2.60]; T3: HR, 3.93 [95% CI 2.91–5.32]; *P* for trend < 0.001) and in the HFmrEF group (T1 vs. T2: HR, 1.38 [95% CI 0.95–2.00]; T3: HR, 1.62 [95% CI 1.08–2.43]; *P* for trend = 0.022), but apparently absent in the HFrEF group (T1 vs. T2: HR, 1.10 [95% CI 0.88–1.38]; T3: HR, 1.22 [95% CI 0.94–1.58]; *P* for trend = 0.142). Finally, we found that the predictive value of the TyG index was more prominent in the HFpEF group, despite taking all-cause death or CV death as an endpoint (both *P* for interaction < 0.001) (Fig. 4).

The dose–response relationships between the TyG index and the risk of the primary endpoints among different subgroups were further elucidated in Additional file 3: Fig. S3. The results indicated that the linear associations between the TyG index and primary outcomes (including all-cause death and CV death) were observed mainly in the MetS group and HFpEF group (all *P* for non-linear association < 0.001).

In addition, we further conducted exploratory analyses among other different subgroups based on the presence of diabetes, hypertension, obesity, dyslipidemia, and ischemic etiology. In the subgroup analyses, the association between the TyG index and the risk of primary outcomes was not materially changed (Additional file 5: Table S5).

### Incremental effect of the TyG index on risk stratification in HF patients

**Fig. 5:**
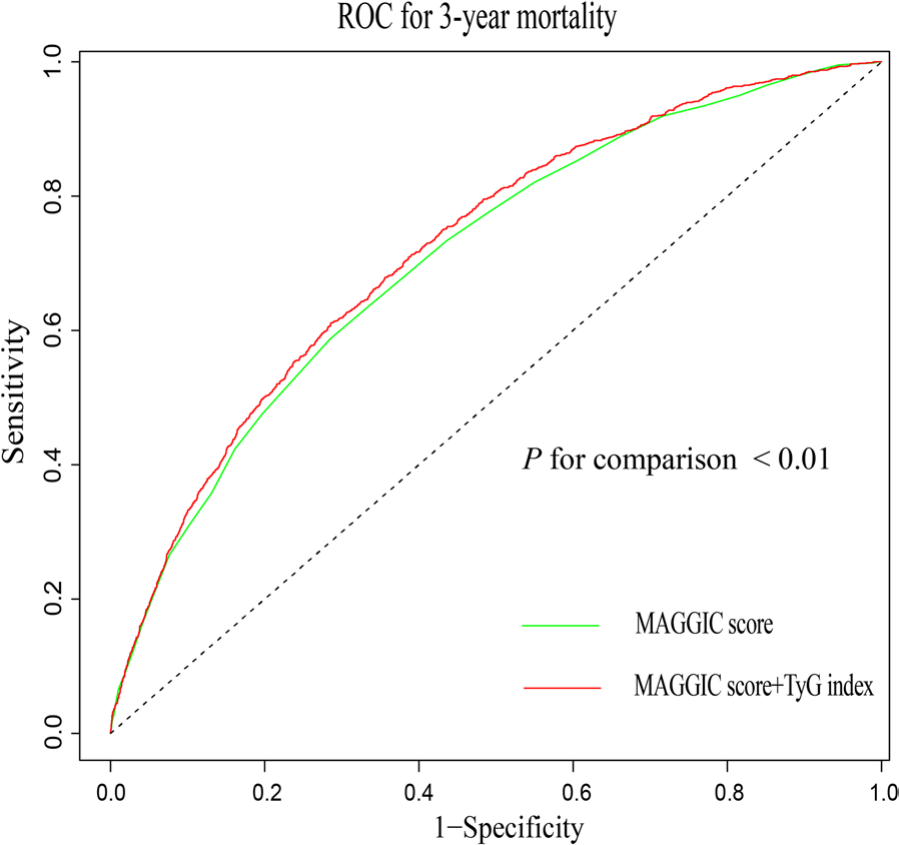
ROC curves of the MAGGIC score and the MAGGIC score plus TyG index for 3-year mortality in HF patients. *ROC curve* receiver operator characteristic curve, *MAGGIC score* Meta-analysis Global Group in Chronic Heart Failure score, *TyG index* triglyceride-glucose index, *HF* heart failure

Finally, whether the TyG index would further increase the predictive ability of the baseline MAGGIC score was evaluated (Fig. 5, Table 3). The addition of the TyG index could slightly, but statistically significantly, improve the area under the receiver operating characteristic curve (AUC) obtained from the MAGGIC score, which consisted of age, sex, systolic blood pressure, BMI, smoking status, LVEF, NYHA class, creatinine, HF duration, diabetes, chronic obstructive pulmonary disorder, and the use of β‑blockers and ACEIs/ARBs (AUC: MAGGIC score, 0.710 [95% CI 0.694–0.726] points vs. MAGGIC score + TyG index, 0.723 [95% CI 0.708–0.738] points; *P* for comparison < 0.01). Moreover, we found that adding the TyG index to the baseline risk score could lead to an increase in the category-free net reclassification index (0.273 [95% CI 0.213–0.334], *P* < 0.01) and integrated discrimination improvement (0.011 [95% CI 0.008–0.015], *P* < 0.01). We further evaluated the incremental effect of the TyG index among different subgroups based on age and sex. The subgroup analyses also showed similar results (Table 3). Decision curve analysis revealed that both the MAGGIC score and the new model (MAGGIC score + TyG index) score had good clinical application value for predicting the 3 year mortality. The net benefit of the new model was superior to the MAGGIC score alone, with a probability range of 0.07–0.36 (Additional file 4: Fig. S4).

**Table 3:** Evaluation the incremental effect of adding the TyG index to the MAGGIC score to predict 3-year mortality

Groups	C-Statistic (95% CI)	*P* value	NRI (95% CI)	*P* value	IDI (95% CI)	*P* value
Overall (n = 6697)						
MAGGIC score	0.710 (0.694–0.726)	Ref	–	Ref	–	Ref
MAGGIC score + TyG	0.723 (0.708–0.738)	** < 0.01**	0.273 (0.213–0.334)	** < 0.01**	0.011 (0.008–0.015)	** < 0.01**
Age < 65 years (n = 3482)						
MAGGIC score	0.691 (0.665–0.716)	Ref	–	Ref	–	Ref
MAGGIC score + TyG	0.706 (0.681–0.731)	** < 0.01**	0.326 (0.230–0.423)	** < 0.01**	0.010 (0.006–0.015)	** < 0.01**
Age ≥ 65 years (n = 3215)						
MAGGIC score	0.674 (0.652–0.695)	Ref	–	Ref	–	Ref
MAGGIC score + TyG	0.689 (0.668–0.710)	** < 0.01**	0.229 (0.151–0.308)	** < 0.01**	0.012 (0.008–0.017)	** < 0.01**
Male (n = 4579)						
MAGGIC score	0.709 (0.690–0.728)	Ref	–	Ref	–	Ref
MAGGIC score + TyG	0.718 (0.699–0.736)	** < 0.01**	0.222 (0.148–0.296)	** < 0.01**	0.008 (0.005–0.011)	** < 0.01**
Female (n = 2118)						
MAGGIC score	0.713 (0.686–0.740)	Ref	–	Ref	–	Ref
MAGGIC score + TyG	0.734 (0.708–0.761)	** < 0.01**	0.353 (0.248–0.458)	** < 0.01**	0.020 (0.013–0.027)	** < 0.01**

*MAGGIC score* Meta-analysis Global Group in Chronic Heart Failure score, *TyG index* triglyceride–glucose index, *NRI* net reclassification improvement, *IDI* integrated discrimination improvement, *CI* confidence interval. *P* values < 0.05 are presented in bold

## Discussion

In the present study, we investigated the association between the TyG index and mortality in CHF patients, and further exploration was also carried out among different subgroups. The results showed that the incidence of mortality significantly increased with the increase of the TyG index. After adjusting for confounding factors, the TyG index was an independent predictor of both all-cause death and CV death. Furthermore, our study revealed that the predictive implication of the TyG index was obvious in different metabolic status groups but more prominent among patients with MetS. Interestingly, the predictive power of the TyG index was mainly found in the non-HFrEF (including HFmrEF and HFpEF) group, not the HFrEF group. Finally, the study demonstrated that adding the TyG index to the baseline risk model may significantly improve the predictive performance for mortality. According to this study, we determined that the TyG index is positively related to increased mortality in CHF patients. Most importantly, this study suggested that a simple method of estimating IR may optimize the risk stratification of mortality in CHF patients.

IR is defined as a decrease in the efficiency of insulin in promoting glucose uptake and utilization, which reflects the disorder of the metabolic balance. IR can lead to CV disease and poor clinical outcomes in various ways, such as inducing endothelial dysfunction, triggering a low-level inflammatory response, and causing systemic glucose–lipid metabolism disorders [21]. Conventional approaches for detecting IR mainly include the hyperinsulinemic–euglycemic clamp technique and HOMA-IR. However, these two methods have common shortcomings, mainly their high cost, complexity, and time-consuming nature, which limits their application in practical clinical settings and large-scale studies [5].

The TyG index, as a novel surrogate marker of IR, has been proven to be strongly related to IR. Fernando et al. [8] found that the TyG index could be used to identify IR with high sensitivity (96.5%) and specificity (85.0%) through hyperinsulinemic–euglycemic clamp testing. At present, the TyG index has been widely used in clinical research with regard to CV disease. Data from a study of 30,291 subjects screened from the China National Diabetes and Metabolic Disorders Study revealed that the TyG index was more suitable for the identification of individuals at high risk of cardiometabolic diseases among the Chinese adult population when compared with other surrogate indices of IR (including triglycerides divided by high-density lipoprotein cholesterol and the metabolic score for IR) [22]. In a retrospective study of 114,603 subjects, Kim et al. found that a higher TyG index could predict a greater risk of cardio-cerebrovascular diseases and all-cause death in men [23]. A study by Íñigo et al. [24] investigated 5014 patients of the Vascular Metabolic CUN cohort during a median period of 10 years and determined that the TyG index might be useful to identify individuals early who are at high risk of developing CV events, including coronary heart disease, cerebrovascular disease, and peripheral arterial disease. Moreover, they demonstrated that adding the TyG index to the Framingham model could significantly improve the predictive power, with AUCs ranging from 0.708 (0.68–0.73) to 0.71 (0.70–0.74) (*P* = 0.014). Ma et al. [25] conducted a study showing that the TyG index was independently and positively associated with adverse CV outcomes, including overall death, non-fatal stroke, non-fatal myocardial infarction, and unplanned repeat revascularization. The above studies have proven that the TyG index, as an easy, effective, and reliable surrogate marker of IR, has good application potential in the CV field.

HF, as the end stage of many diseases, is a complex clinical syndrome with multiple potential and complex mechanisms. Many studies have shown that the occurrence and development of HF are closely related to IR. On the one hand, CHF may cause or exacerbate the degree of IR. CHF can trigger the disorder of the neuroendocrine system of the body, including hyperactivation of the sympathetic nerve and elevated levels of inflammatory cytokines. A heightened adrenergic drive or increased levels of tumor necrosis factor-α may contribute to increases in free fatty acid oxidation and subsequent IR [26]. Meanwhile, the overstimulation of β-adrenergic receptors weakens insulin sensitivity through an Akt-mediated effect [27]. Ciccarelli et al. showed that ischemia-induced up-regulation of G protein-coupled receptor kinase 2 causes IR by interrupting insulin signaling [28]. On the other hand, IR in turn may trigger or aggravate the extent of CHF. Vardeny et al. [29] prospectively analyzed 12,606 participants from the Atherosclerosis Risk in Communities study using HOMA-IR to assess the relationship between IR and incident HF and found that the degree of IR (defined by levels of HOMA-IR) was positively correlated with the risk of incident HF. Consistent with the above research results, Banerjee et al. found from a 12-year study that the fasting insulin level was positively associated with the risk of subsequent HF, even in subjects without CV disease [30]. A retrospective cohort study based on a Chinese population (138,620 participants) revealed that the TyG index was positively related to a high risk of developing HF in a J-shaped dose–response relationship [31].

At present, there are relatively few studies on the relationship between the TyG index and long-term CHF prognosis. It has been reported that impaired insulin sensitivity is associated with the severity of CHF in terms of reduced peak oxygen uptake, NYHA class, and the 6 min walk test [32]. Yang et al. evaluated the relationship between the TyG index and myocardial fibrosis, which was calculated by measuring extracellular volume fraction during CV magnetic resonance examination. Their study indicated that TyG index could be a novel biomarker of myocardial fibrosis and was independently associated with primary outcomes (including all-cause death and HF hospitalization). Moreover, in a retrospective study of 546 patients diagnosed with CHF and type 2 diabetes [33], Guo et al. revealed that there was a positive association between the TyG index and adverse outcomes (including CV death and rehospitalization due to HF). Consistent with previous findings, our study enrolled 6697 CHF patients with a median follow-up of 3.9 years and found that a higher level of the TyG index was significantly related to a greater risk of mortality, which remained apparent even after adjusting for multiple factors. The association between the TyG index and adverse clinical outcomes may be explained as follows. First, IR can reduce the bioavailability of glucose and improve a shift to fatty acid metabolism, leading to an increase in myocardial oxygen consumption and a reduction in the compensatory capacity of the myocardium [33–35]. Second, glycolipid metabolism disorder can trigger an increase in reactive oxygen species, mitochondrial dysfunction, endoplasmic reticulum stress, impaired cardiac calcium signaling, systemic low-grade inflammation, and inappropriate activation of the renin–angiotensin system, exacerbating the progression of HF [32, 36, 37]. Third, the deposition of glycosylation end products can increase diastolic left ventricular stiffness and inactivate nitric oxide, which is a protective factor for the vascular endothelium [38]. Finally, the vicious cycle between CHF and IR may also exacerbate the deterioration of cardiac function [26].

Given that IR is the core of MetS and there is great heterogeneity among different HF phenotypes, we further analyzed the predictive power of the TyG index among different metabolic status groups and different HF phenotype groups, which other studies have not attempted. For the first time, our study showed that, while the TyG index had significant potential for predicting adverse clinical outcomes in different metabolic status groups, this phenomenon was more pronounced in the MetS group. More interestingly, when the analysis was stratified by HF phenotype, we found that this prognostic relationship only existed in the non-HFrEF (HFmrEF and HFpEF) group, and not in the HFrEF group. Several explanations may account for the above differences: first, IR is the main feature of MetS and is a more serious condition in this patient population than IR in the non-MetS group. IR can mediate myocardial damage through a variety of pathways, such as endothelial dysfunction, abnormal fatty acid metabolism, production of glycosylated end-products and free radicals, and overactivation of the renin–angiotensin system [34, 36]. Second, HFrEF is mostly caused by heart disease [39], including coronary heart disease, myocardiopathy, valvulopathy, and arrhythmias. The effect of extracardiac causes, including IR, may be relatively weak. Third, IR, as one of the main causes of HFpEF, can not only trigger but also aggravate the development of HFpEF through unique pathogenic mechanisms, such as the increase in epicardial adipose tissue [40] and inducing concentric left ventricular remodeling [41]. Fourth, the effect of the TyG index on the prognosis of HF patients was more prominent in the MetS group, which was confirmed by our study. What is more, extracardiac comorbidities (including obesity, hypertension, diabetes, and MetS) affect a high proportion of HFpEF patients [42, 43]. Finally, although it is suggested that HFmrEF may be a transitional state between HFpEF and HFrEF [44], patients with HFmrEF have clinical features and prognoses that are more similar to those of patients with HFpEF than those of patients with HFrEF [45]. In addition, another novelty of our research is that adding TyG index to the established risk model led to a significant incremental effect on the predictive accuracy for mortality.

## Limitations

There are several limitations worth considering in this study. First, due to the lack of serum insulin level measurements, we could not calculate HOMA-IR values and compare them to TyG indices. Second, although the sample size of this study was large, only baseline measurements at admission were available, and data collected at different time points after discharge were lacking. Third, a certain degree of recall bias or reporting bias may occur during follow-up. Fourth, because of the inherent nature of observational research, unmeasured confounding factors may have affected the results of the study. In this respect, our results should be interpreted with caution. Fifth, this was an observational study, and causal relationships between the exposures and study outcomes could not be established. Finally, although our study showed that the TyG index had certain prognostic significance for patients with CHF, its practical clinical application value remains to be further confirmed by prospective studies.

## Conclusions

In conclusion, the current study demonstrated that the TyG index is strongly associated with the risk of mortality in CHF patients, especially those with MetS and those in the HFpEF group. In addition, adding the TyG index to a baseline risk model had an incremental effect on the predictive value for mortality.

Therefore, routinely monitoring the TyG index during the management of patients with CHF might contribute to refining risk stratification.

### Supplementary Information

**Additional file 1: Fig S1. **HRs for all-cause death and CV death in heart failure patients using spline analyses adjusted for model 2. Restricted cubic splines of the TyG index with three knots were used. Red lines represented references for hazard ratios, and red areas represented 95% confidence intervals.

**Additional file 2: Fig S2. **Kaplan–Meier estimation of all-cause death and CV death by tertiles of TyG index among different subgroups: all-cause death in MetS group, CV death in MetS group, all-cause death in non-MetS group, CV death in non-MetS group, all-cause death in HFrEF group, CV death in HFrEF group, all-cause death in HFmrEF group, CV death in HFmrEF group, all-cause death in HFpEF group, and CV death in HFpEF group.

**Additional file 3: Fig S3. **HRs for all-cause death and CV death using spline analyses adjusted for model 2 among different subgroups: all-cause death in MetS group, CV death in MetS group, all-cause death in non-MetS group, CV death in non-MetS group, all-cause death in HFrEF group, CV death in HFrEF group, all-cause death in HFmrEF group, CV death in HFmrEF group, all-cause death in HFpEF group, and CV death in HFpEF group. Restricted cubic splines of the TyG index with three knots were used. Red lines represented references for hazard ratios, and red areas represented 95% confidence intervals.

**Additional file 4: Fig S4. **The decision curve analysis comparing the MAGGIC score and its combination with the TyG index.

**Additional file 5: Table S1. **Baseline characteristics of the study population according to TyG index tertiles after PSM analysis. **Table S2. **HRs of primary outcomes according to TyG index tertiles after PSM analysis. **Table S3. **HRs of primary outcomes according to TyG index tertiles in different metabolic status groups. **Table S4. **HRs of primary outcomes according to TyG index tertiles in different heart failure phenotypes. **Table S5. **HRs of primary outcomes according to TyG index tertiles among other different subgroups.

## Data Availability

The datasets used and analyzed during the current study are available from the corresponding author on reasonable request.

## Abbreviations

CHF: Chronic heart failure
TyG index: Triglyceride–glucose index
IR: Insulin resistance
CV death: Cardiovascular death
HOMA-IR: Homeostasis model assessment of insulin resistance
MAGGIC: Meta-analysis global group in chronic heart failure
CTD: Connective tissue diseases
BMI: Body mass index
SBP: Systolic blood pressure
DBP: Diastolic blood pressure
HR: Heart rate
LVEF: Left ventricular ejection fraction
NYHA: New York heart association
AF: Atrial fibrillation
CKD: Chronic kidney disease
MI: Myocardial infarction
PAD: Peripheral arterial disease
COPD: Chronic obstructive pulmonary disease
MetS: Metabolic syndrome
PCI: Percutaneous coronary intervention
CABG: Coronary artery bypass grafting
WBC: White blood cell
ALT: Alanine aminotransferase
AST: Aspartate aminotransferase
TBil: Total bilirubin
BUN: Blood urea nitrogen
eGFR: Estimated glomerular filtration rate
FBG: Fasting blood glucose
TG: Triglyceride
TC: Total cholesterol
LDL-C: Low-density lipoprotein cholesterol
HDL-C: High-density lipoprotein cholesterol
LDH: Lactic dehydrogenase
cTnT: Cardiac troponin T
NT-proBNP: N-terminal pro-brain natriuretic peptide
ACEI/ARB: Angiotensin converting enzyme inhibitor/angiotensin receptor blocker
ARNI: Angiotensin receptor-neprilysin inhibitors
CCB: Calcium channel blockers
SGLT2 inhibitors: Sodium-glucose co-transporter-2 inhibitors
CI: Confidence interval
HR: Hazard ratio
PSM: Propensity score matching
HFrEF: Heart failure with reduced ejection fraction
HFmrEF: Heart failure with mildly reduced ejection fraction
HFpEF: Heart failure with preserved ejection fraction
NRI: Net reclassification improvement
IDI: Integrated discrimination improvement
ROC curve: Receiver operator characteristic curve
AUC: Area under the receiver operating characteristic curve
DCA: Decision curve analysis

## References

[1] James SL, Abate D, Abate KH, Abay SM, Abbafati C, Abbasi N, Abbastabar H, Abd-Allah F, Abdela J, Abdelalim A (2018). Global, regional, and national incidence, prevalence, and years lived with disability for 354 diseases and injuries for 195 countries and territories, 1990–2017: a systematic analysis for the global burden of disease study 2017. Lancet.

[2] Perrone-Filardi P, Savarese G, Scarano M, Cavazzina R, Trimarco B, Minneci S, Maggioni AP, Tavazzi L, Tognoni G, Marchioli R (2015). Prognostic impact of metabolic syndrome in patients with chronic heart failure: data from GISSI-HF trial. Int J Cardiol.

[3] Paolillo S, Rengo G, Pellegrino T, Formisano R, Pagano G, Gargiulo P, Savarese G, Carotenuto R, Petraglia L, Rapacciuolo A (2015). Insulin resistance is associated with impaired cardiac sympathetic innervation in patients with heart failure. Eur Heart J Cardiovasc Imaging.

[4] DeFronzo RA, Tobin JD, Rowe JW, Andres R (1978). Glucose intolerance in uremia quantification of pancreatic beta cell sensitivity to glucose and tissue sensitivity to insulin. J Clin Invest.

[5] Muniyappa R, Lee S, Chen H, Quon MJ (2008). Current approaches for assessing insulin sensitivity and resistance in vivo: advantages, limitations, and appropriate usage. Am J Physiol Endocrinol Metab.

[6] Mohd Nor NS, Lee S, Bacha F, Tfayli H, Arslanian S (2016). Triglyceride glucose index as a surrogate measure of insulin sensitivity in obese adolescents with normoglycemia, prediabetes, and type 2 diabetes mellitus: comparison with the hyperinsulinemic-euglycemic clamp. Pediatr Diabetes.

[7] Vasques AC, Novaes FS, de Oliveira MS, Souza JR, Yamanaka A, Pareja JC, Tambascia MA, Saad MJ, Geloneze B (2011). TyG index performs better than HOMA in a Brazilian population: a hyperglycemic clamp validated study. Diabetes Res Clin Pract.

[8] Guerrero-Romero F, Simental-Mendia LE, Gonzalez-Ortiz M, Martinez-Abundis E, Ramos-Zavala MG, Hernandez-Gonzalez SO, Jacques-Camarena O, Rodriguez-Moran M (2010). The product of triglycerides and glucose, a simple measure of insulin sensitivity comparison with the euglycemic-hyperinsulinemic clamp. J Clin Endocrinol Metab.

[9] Yang S, Du Y, Liu Z, Zhang R, Lin X, Ouyang Y, Chen H (2021). Triglyceride-glucose index and extracellular volume fraction in patients with heart failure. Front Cardiovasc Med.

[10] Lee EY, Yang HK, Lee J, Kang B, Yang Y, Lee SH, Ko SH, Ahn YB, Cha BY, Yoon KH (2016). Triglyceride glucose index, a marker of insulin resistance, is associated with coronary artery stenosis in asymptomatic subjects with type 2 diabetes. Lipids Health Dis.

[11] Liu X, Tan Z, Huang Y, Zhao H, Liu M, Yu P, Ma J, Zhao Y, Zhu W, Wang J (2022). Relationship between the triglyceride-glucose index and risk of cardiovascular diseases and mortality in the general population: a systematic review and meta-analysis. Cardiovasc Diabetol.

[12] McDonagh TA, Metra M, Adamo M, Gardner RS, Baumbach A, Bohm M, Burri H, Butler J, Celutkiene J, Chioncel O (2021). 2021 ESC Guidelines for the diagnosis and treatment of acute and chronic heart failure. Eur Heart J.

[13] Li P, Zhao H, Zhang J, Ning Y, Tu Y, Xu D, Zeng Q (2021). Similarities and differences between HFmrEF and HFpEF. Front Cardiovasc Med.

[14] L AS, S LA, S CH, Z YL, C AF, F HI, K JW, E P, Van L F, G T, C J (2009). A new equation to estimate glomerular filtration rate. Ann Intern Med.

[15] de Boer IH, Khunti K, Sadusky T, Tuttle KR, Neumiller JJ, Rhee CM, Rosas SE, Rossing P, Bakris G (2022). Diabetes management in chronic kidney disease: a consensus report by the American diabetes association (ADA) and kidney disease: improving global outcomes (KDIGO). Diabetes Care.

[16] Criqui MH, Aboyans V (2015). Epidemiology of peripheral artery disease. Circ Res.

[17] Pocock SJ, Ariti CA, McMurray JJ, Maggioni A, Kober L, Squire IB, Swedberg K, Dobson J, Poppe KK, Whalley GA (2013). Predicting survival in heart failure: a risk score based on 39 372 patients from 30 studies. Eur Heart J.

[18] Pan X-F, Wang L, Pan A (2021). Epidemiology and determinants of obesity in China. Lancet Diabetes Endocrinol.

[19] Jia W, Weng J, Zhu D, Ji L, Lu J, Zhou Z, Zou D, Guo L, Ji Q, Chen L (2019). Standards of medical care for type 2 diabetes in China 2019. Diabetes Metab Res Rev.

[20] Desquilbet L, Mariotti F (2010). Dose-response analyses using restricted cubic spline functions in public health research. Stat Med.

[21] Ormazabal V, Nair S, Elfeky O, Aguayo C, Salomon C, Zuñiga FA (2018). Association between insulin resistance and the development of cardiovascular disease. Cardiovasc Diabetol.

[22] Yu X, Wang L, Zhang W, Ming J, Jia A, Xu S, Li Q, Ji Q (2019). Fasting triglycerides and glucose index is more suitable for the identification of metabolically unhealthy individuals in the Chinese adult population: a nationwide study. J Diabetes Invest.

[23] Kim J, Shin SJ, Kang HT (2021). The association between triglyceride-glucose index, cardio-cerebrovascular diseases, and death in Korean adults: a retrospective study based on the NHIS-HEALS cohort. PLoS ONE.

[24] Sánchez-Íñigo L, Navarro-González D, Fernández-Montero A, Pastrana-Delgado J, Martínez JA (2016). The TyG index may predict the development of cardiovascular events. Eur J Clin Invest.

[25] Ma X, Dong L, Shao Q, Cheng Y, Lv S, Sun Y, Shen H, Wang Z, Zhou Y, Liu X (2020). Triglyceride glucose index for predicting cardiovascular outcomes after percutaneous coronary intervention in patients with type 2 diabetes mellitus and acute coronary syndrome. Cardiovasc Diabetol.

[26] Li C, Ford ES, McGuire LC, Mokdad AH (2007). Association of metabolic syndrome and insulin resistance with congestive heart failure: findings from the third national health and nutrition examination survey. J Epidemiol Community Health.

[27] Morisco C, Condorelli G, Trimarco V, Bellis A, Marrone C, Condorelli G, Sadoshima J, Trimarco B (2005). Akt mediates the cross-talk between beta-adrenergic and insulin receptors in neonatal cardiomyocytes. Circ Res.

[28] Ciccarelli M, Chuprun JK, Rengo G, Gao E, Wei Z, Peroutka RJ, Gold JI, Gumpert A, Chen M, Otis NJ (2011). G protein-coupled receptor kinase 2 activity impairs cardiac glucose uptake and promotes insulin resistance after myocardial ischemia. Circulation.

[29] Vardeny O, Gupta DK, Claggett B, Burke S, Shah A, Loehr L, Rasmussen-Torvik L, Selvin E, Chang PP, Aguilar D (2013). Insulin resistance and incident heart failure the ARIC study (atherosclerosis risk in communities). JACC Heart Fail.

[30] Banerjee D, Biggs ML, Mercer L, Mukamal K, Kaplan R, Barzilay J, Kuller L, Kizer JR, Djousse L, Tracy R (2013). Insulin resistance and risk of incident heart failure: cardiovascular health study. Circ Heart Fail.

[31] Xu L, Wu M, Chen S, Yang Y, Wang Y, Wu S, Tian Y (2022). Triglyceride-glucose index associates with incident heart failure: a cohort study. Diabetes Metab.

[32] Doehner W, Rauchhaus M, Ponikowski P, Godsland IF, von Haehling S, Okonko DO, Leyva F, Proudler AJ, Coats AJ, Anker SD (2005). Impaired insulin sensitivity as an independent risk factor for mortality in patients with stable chronic heart failure. J Am Coll Cardiol.

[33] Guo W, Zhao L, Mo F, Peng C, Li L, Xu Y, Guo W, Sun A, Yan H, Wang L (2021). The prognostic value of the triglyceride glucose index in patients with chronic heart failure and type 2 diabetes: a retrospective cohort study. Diabetes Res Clin Pract.

[34] Aroor AR, Mandavia CH, Sowers JR (2012). Insulin resistance and heart failure: molecular mechanisms. Heart Fail Clin.

[35] Zheng L, Li B, Lin S, Chen L, Li H (2019). Role and mechanism of cardiac insulin resistance in occurrence of heart failure caused by myocardial hypertrophy. Aging.

[36] Jia G, Hill MA, Sowers JR (2018). Diabetic cardiomyopathy: an update of mechanisms contributing to this clinical entity. Circ Res.

[37] Laakso M, Kuusisto J (2014). Insulin resistance and hyperglycaemia in cardiovascular disease development. Nat Rev Endocrinol.

[38] van Heerebeek L, Hamdani N, Handoko ML, Falcao-Pires I, Musters RJ, Kupreishvili K, Ijsselmuiden AJ, Schalkwijk CG, Bronzwaer JG, Diamant M (2008). Diastolic stiffness of the failing diabetic heart: importance of fibrosis, advanced glycation end products, and myocyte resting tension. Circulation.

[39] Packer M (2019). Drugs that ameliorate epicardial adipose tissue inflammation may have discordant effects in heart failure with a preserved ejection fraction as compared with a reduced ejection fraction. J Cardiac Fail.

[40] Liu J, Yu Q, Li Z, Zhou Y, Liu Z, You L, Tao L, Dong Q, Zuo Z, Gao L (2023). Epicardial adipose tissue density is a better predictor of cardiometabolic risk in HFpEF patients: a prospective cohort study. Cardiovasc Diabetol.

[41] Velagaleti RS, Gona P, Chuang ML, Salton CJ, Fox CS, Blease SJ, Yeon SB, Manning WJ, O'Donnell CJ (2010). Relations of insulin resistance and glycemic abnormalities to cardiovascular magnetic resonance measures of cardiac structure and function: the framingham heart study. Circ Cardiovasc Imaging.

[42] Yoon HJ, Ahn Y, Kim KH, Park JC, Choi DJ, Han S, Jeon ES, Cho MC, Kim JJ, Yoo BS (2013). The prognostic implication of metabolic syndrome in patients with heart failure. Korean Circ J.

[43] Owan TE, Hodge DO, Herges RM, Jacobsen SJ, Roger VL, Redfield MM (2006). Trends in prevalence and outcome of heart failure with preserved ejection fraction. N Engl J Med.

[44] Tsuji K, Sakata Y, Nochioka K, Miura M, Yamauchi T, Onose T, Abe R, Oikawa T, Kasahara S, Sato M (2017). Characterization of heart failure patients with mid-range left ventricular ejection fraction-a report from the CHART-2 study. Eur J Heart Fail.

[45] Hsu JJ, Ziaeian B, Fonarow GC (2017). Heart failure with mid-range (borderline) ejection fraction: clinical implications and future directions. JACC Heart Failure.

