# Correction to: Association between the triglyceride-glucose index and the risk of mortality among patients with chronic heart failure: results from a retrospective cohort study in China

**DOI:** 10.1186/s12933-023-01978-2

**Published:** 2023-09-15

**Authors:** You Zhou, Chi Wang, Hebin Che, Liting Cheng, Di Zhu, Chongyou Rao, Qin Zhong, Zongren Li, Xiao Wang, Zisheng Wu, Kunlun He

**Affiliations:** 1 School of Medicine, Nankai University, No.94 Weijin Road, Nankai District, Tianjin, 300071 China; 2 Medical Big Data Research Center, Medical Innovation Research Department of PLA General Hospital, No.28 Fuxing Road, Haidian District, Beijing, 100853 China; 3 Chinese PLA General Hospital and Medical School, No.28 Fuxing Road, Haidian District, Beijing, 100853 China

**Correction to: Cardiovascular Diabetology (2023) 22:171**

10.1186/s12933-023-01895-4

Following publication of the original article [1], the authors further explained the definition of the study population (chronic heart failure, CHF). In this study, CHF was defined according to the 2021 European Society for Cardiology Guidelines for the Diagnosis and Treatment of Acute and Chronic Heart Failure, which did not include patients with acute exacerbation of CHF (AE-CHF), who tended to have lower LVEF levels (≤ 40%) on admission.

In addition, the authors submitted a corrected version of Additional file 1: Table S5, as the original version contained a mistake. In exploratory analyses, the association between the TyG index and the risk of primary outcomes remained obvious in all subgroups (all *P* for trend < 0.001, Additional file 1: Table S5). While the updated exploratory analyses revealed stronger correlations between the TyG index and all-cause death in patients with hypertension and those with non-ischemic etiology, the main reason for such phenomenon was still attributed to the higher proportion of HFpEF in these patients.

In Statistical analysis section, the proper description of the linear trends analysis, with quartiles corrected to tertiles, is “The linear trends across TyG tertiles were evaluated by a median value within each tertile as a continuous variable.”

In Discussion section, the reference for “*Yang et al. evaluated the relationship between the TyG index and myocardial fibrosis, which was calculated by measuring extracellular volume fraction during CV magnetic resonance examination.*” should be cited here as Yang et al. (2021).

Such mild changes did not cause substantial influence to the main conclusion and clinical significance of our study, that is, the TyG index is strongly associated with the risk of mortality in CHF patients.

Yang S, Du Y, Liu Z, Zhang R, Lin X, Ouyang Y, Chen H: Triglyceride-Glucose Index and Extracellular Volume Fraction in Patients With Heart Failure. Front Cardiovasc Med. 2021;8:704462

### Electronic Supplementary Material

Below is the link to the electronic supplementary material

Supplementary Material 1

## References

[1] Zhou Y, Wang C, Che H (2023). Association between the triglyceride–glucose index and the risk of mortality among patients with chronic heart failure: results from a retrospective cohort study in China. Cardiovasc Diabetol.

