# Supplementary figures and images for: Association between the triglyceride–glucose index and the risk of mortality among patients with chronic heart failure: results from a retrospective cohort study in China
Source: Cardiovasc Diabetol. 2023 Jul 7;22:171. doi: 10.1186/s12933-023-01895-4 (PMC10329381; doi:10.1186/s12933-023-01895-4)

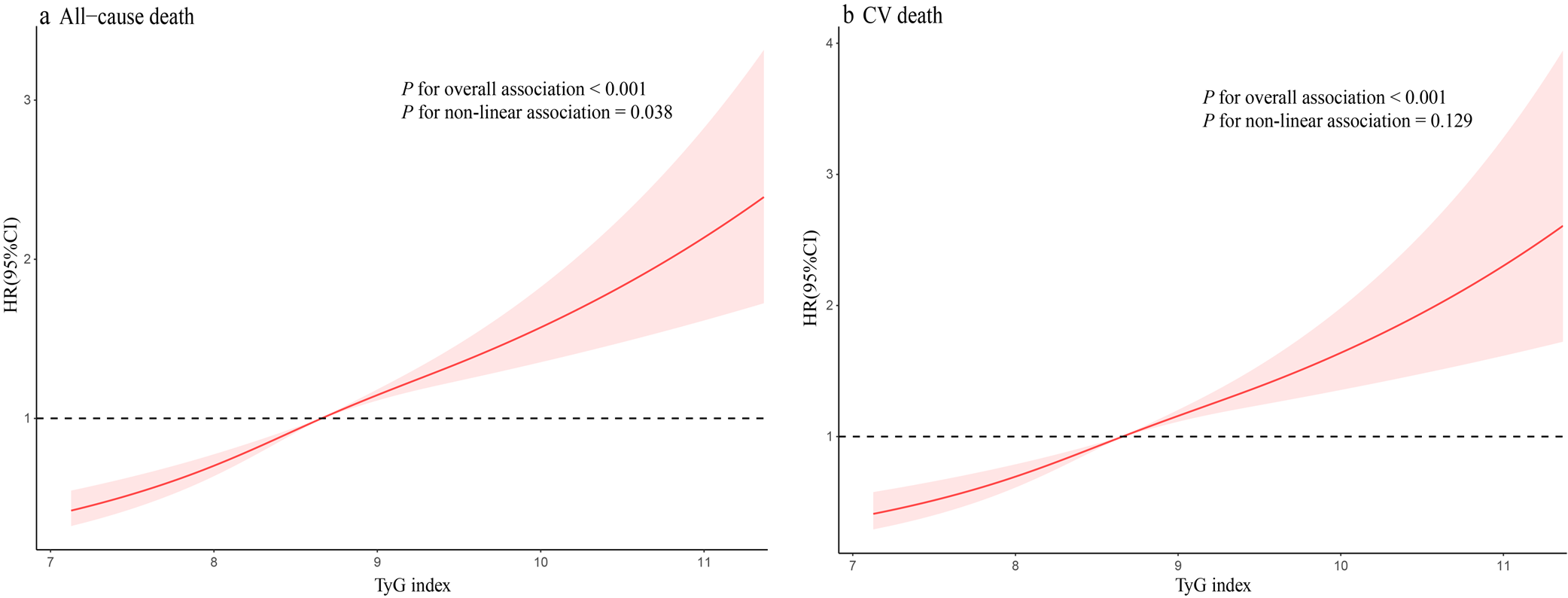

Supplement: Supplementary file 1 — Additional file 1: Fig S1. HRs for all-cause death and CV death in heart failure patients using spline analyses adjusted for model 2. Restricted cubic splines of the TyG index with three knots were used. Red lines represented references for hazard ratios, and red areas represented 95% confidence intervals. [file 12933_2023_1895_MOESM1_ESM.tif]

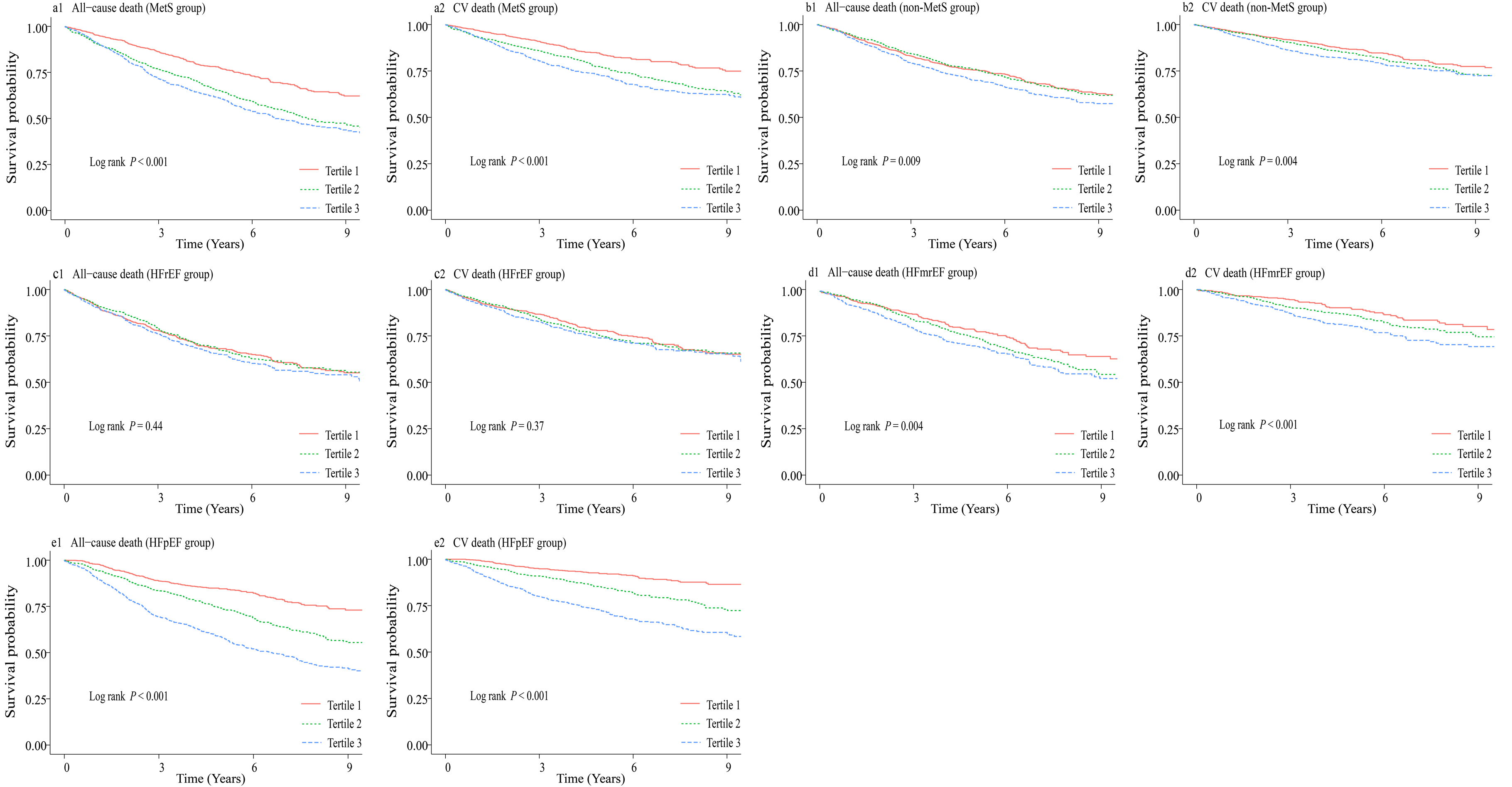

Supplement: Supplementary file 2 — Additional file 2: Fig S2. Kaplan–Meier estimation of all-cause death and CV death by tertiles of TyG index among different subgroups: all-cause death in MetS group, CV death in MetS group, all-cause death in non-MetS group, CV death in non-MetS group, all-cause death in HFrEF group, CV death in HFrEF group, all-cause death in HFmrEF group, CV death in HFmrEF group, all-cause death in HFpEF group, and CV death in HFpEF group. [file 12933_2023_1895_MOESM2_ESM.tif]

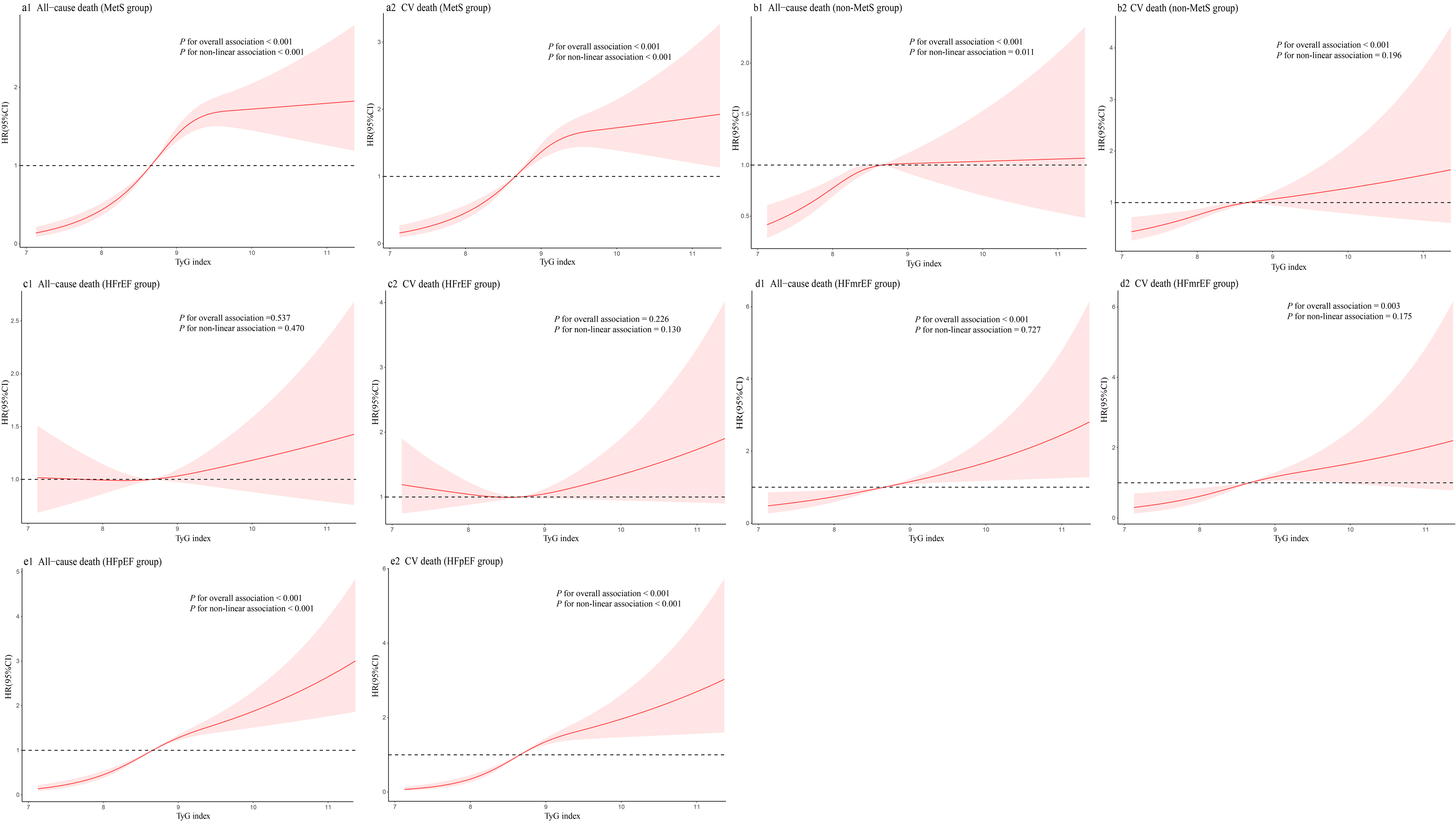

Supplement: Supplementary file 3 — Additional file 3: Fig S3. HRs for all-cause death and CV death using spline analyses adjusted for model 2 among different subgroups: all-cause death in MetS group, CV death in MetS group, all-cause death in non-MetS group, CV death in non-MetS group, all-cause death in HFrEF group, CV death in HFrEF group, all-cause death in HFmrEF group, CV death in HFmrEF group, all-cause death in HFpEF group, and CV death in HFpEF group. Restricted cubic splines of the TyG index with three knots were used. Red lines represented references for hazard ratios, and red areas represented 95% confidence intervals. [file 12933_2023_1895_MOESM3_ESM.tif]

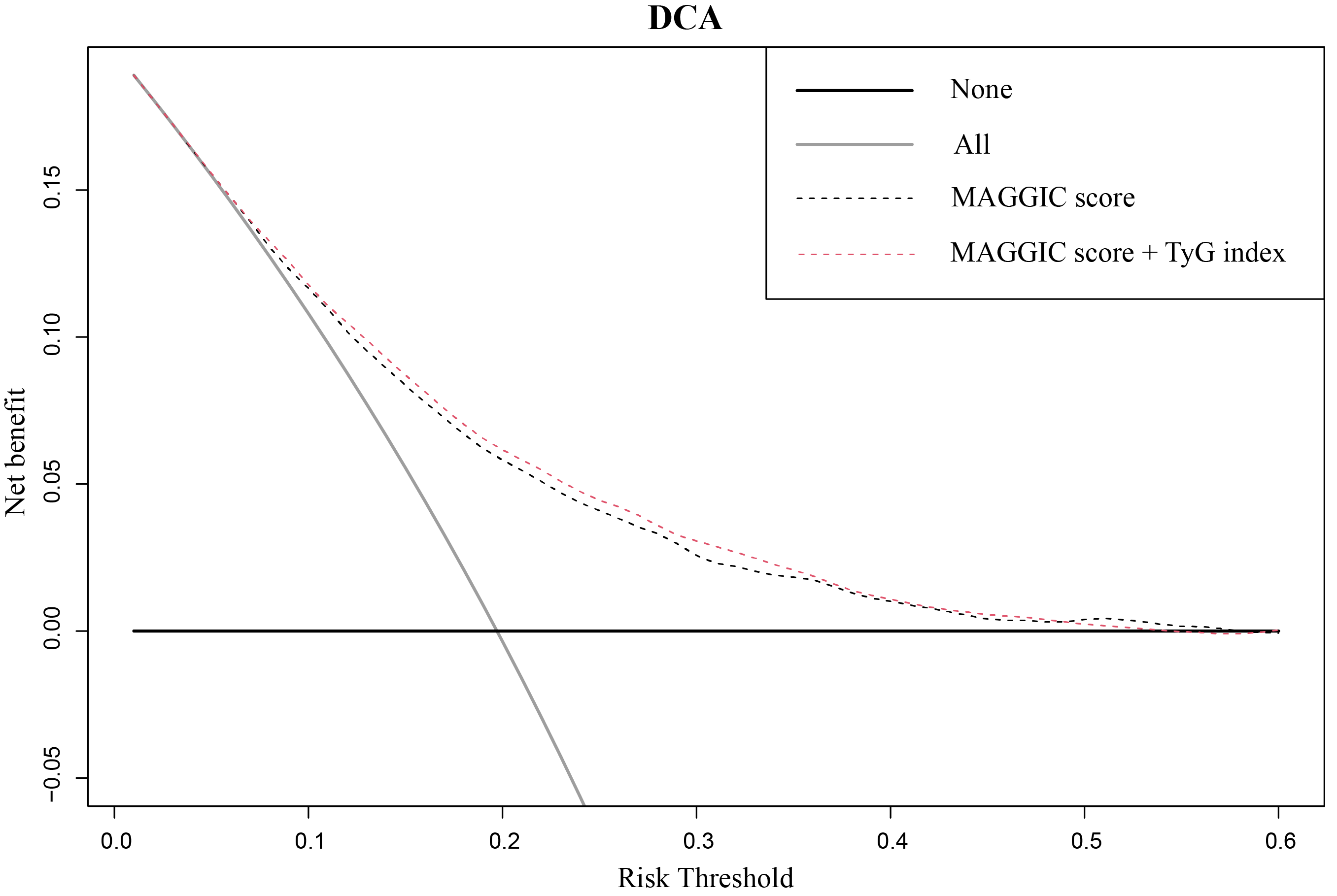

Supplement: Supplementary file 4 — Additional file 4: Fig S4. The decision curve analysis comparing the MAGGIC score and its combination with the TyG index. [file 12933_2023_1895_MOESM4_ESM.tif]
