# Supplementary material for: Association between the triglyceride–glucose index and the risk of mortality among patients with chronic heart failure: results from a retrospective cohort study in China
Source: Cardiovasc Diabetol. 2023 Jul 7;22:171. doi: 10.1186/s12933-023-01895-4 (PMC10329381; doi:10.1186/s12933-023-01895-4)
Supplement: Supplementary file 5 — Additional file 5: Table S1. Baseline characteristics of the study population according to TyG index tertiles after PSM analysis. Table S2. HRs of primary outcomes according to TyG index tertiles after PSM analysis. Table S3. HRs of primary outcomes according to TyG index tertiles in different metabolic status groups. Table S4. HRs of primary outcomes according to TyG index tertiles in different heart failure phenotypes. Table S5. HRs of primary outcomes according to TyG index tertiles among other different subgroups. [file 12933_2023_1895_MOESM5_ESM.docx]

| **Table S1 Baseline characteristics of the study population according to TyG index tertiles after PSM analysis** | | | | | |
| --- | --- | --- | --- | --- | --- |
| **Variables** | **Total** | **Tertile of TyG index** | | | ***P* value** |
|  |  | **T1** | **T2** | **T3** |  |
|  | **n=3021** | **n=1007** | **n=1007** | **n=1007** |  |
| TyG index | 8.68 ± 0.59 | < 8.40 | 8.40-8.93 | ≥ 8.93 | - |
| Demographics |  |  |  |  |  |
| Age (years) | 64.2 (54.1-73.8) | 64.8 (54.5-73.8) | 64.4 (55.0-73.8) | 63.7 (53.2-74.0) | 0.632 |
| Male (%) | 2104 (69.65%) | 685 (68.02%) | 725 (72.00%) | 694 (68.92%) | 0.126 |
| BMI (kg/m^2^) | 25.3 (22.9-28.1) | 24.4 (22.1-27.2) | 25.4 (22.9-28.1) | 26.1 (23.5-28.6) | **<0.001** |
| Medical measurements |  |  |  |  |  |
| SBP (mmHg) | 130.0 (116.0-145.0) | 130.0 (116.0-145.0) | 129.0 (116.0-144.0) | 129.0 (116.0-144.0) | 0.880 |
| DBP (mmHg) | 75.0 (66.0-84.0) | 74.0 (65.0-84.0) | 75.0 (67.0-85.0) | 76.0 (66.0-85.0) | 0.059 |
| HR (bpm) | 78.0 (70.0-90.0) | 77.0 (69.0-90.0) | 79.0 (70.0-90.0) | 80.0 (70.0-92.0) | **0.005** |
| Smoking (%) |  |  |  |  | **0.014** |
| Current smoker | 760 (25.16%) | 217 (21.55%) | 267 (26.51%) | 276 (27.41%) |  |
| Former smoker | 613 (20.29%) | 214 (21.25%) | 189 (18.77%) | 210 (20.85%) |  |
| Never smoker | 1648 (54.55%) | 576 (57.20%) | 551 (54.72) | 521 (51.74%) |  |
| Drinking (%) |  |  |  |  | 0.149 |
| Current drinker | 734 (24.30%) | 229 (22.74%) | 242 (24.03%) | 263 (26.12%) |  |
| Former drinker | 300 (9.93%) | 104 (10.33%) | 87 (8.64%) | 109 (10.82%) |  |
| Never drinker | 1987 (65.77%) | 674 (66.93%) | 678 (67.33%) | 635 (63.06%) |  |
| LVEF (%) |  |  |  |  | 0.116 |
| ≤ 40% | 941 (31.15%) | 314 (31.18%) | 336 (33.37%) | 291 (28.90%) |  |
| 41%~49% | 681 (22.54%) | 233 (23.14%) | 230 (22.84%) | 218 (21.65%) |  |
| ≥ 50% | 1399 (46.31%) | 460 (45.68%) | 441 (43.79%) | 498 (49.45%) |  |
| NYHA classification (%) |  |  |  |  | 0.818 |
| I-II | 1352 (44.75%) | 445 (44.19%) | 446 (44.29%) | 461 (45.78%) |  |
| III | 1296 (42.90%) | 443 (43.99%) | 429 (42.60%) | 424 (42.11%) |  |
| IV | 373 (12.35%) | 119 (11.82%) | 132 (13.11%) | 122 (12.12%) |  |
| Medical history (%) |  |  |  |  |  |
| Hypertension | 1996 (66.07%) | 666 (66.14%) | 673 (66.83%) | 657 (65.24%) | 0.752 |
| Diabetes | 1465 (48.49%) | 485 (48.16%) | 491 (48.76%) | 489 (48.56%) | 0.964 |
| AF | 934 (30.92%) | 328 (32.57%) | 312 (30.98%) | 294 (29.20%) | 0.261 |
| CKD (Stages III-IV) | 801 (26.51%) | 269 (26.71%) | 252 (25.02%) | 280 (27.81%) | 0.363 |
| Previous MI | 1065 (35.25%) | 351 (34.86%) | 382 (37.93%) | 332 (32.97%) | 0.063 |
| Angina | 942 (31.18%) | 305 (30.29%) | 327 (32.47%) | 310 (30.78%) | 0.540 |
| Stroke | 583 (19.30%) | 199 (19.76%) | 193 (19.17%) | 191 (18.97%) | 0.895 |
| PAD | 468 (15.49%) | 165 (16.39%) | 145 (14.40%) | 158 (15.69%) | 0.458 |
| COPD | 120 (3.97%) | 31 (3.08%) | 40 (3.97%) | 49 (4.87%) | 0.121 |
| Previous heart surgery (%) |  |  |  |  |  |
| PCI | 624 (20.66%) | 212 (21.05%) | 224 (22.24%) | 188 (18.67%) | 0.131 |
| CABG | 136 (4.50%) | 54 (5.36%) | 48 (4.77%) | 34 (3.38%) | 0.088 |
| Cardiac valve surgery | 101 (3.34%) | 37 (3.67%) | 42 (4.17%) | 22 (2.18%) | **0.036** |
| Pacemaker therapy | 126 (4.17%) | 38 (3.77%) | 41 (4.07%) | 47 (4.67%) | 0.594 |
| **Table S1 (continued)** |  |  |  |  |  |
| **Variables** | **Total** | **T1** | **T2** | **T3** | ***P* value** |
| Laboratory measurements |  |  |  |  |  |
| WBC (10^9^/L) | 6.61 (5.40-8.11) | 6.14 (5.09-7.49) | 6.64 (5.38-8.03) | 7.10 (5.96-8.63) | **<0.001** |
| Hemoglobin (g/L) | 136.0 (121.0-149.0) | 134.0 (117.0-146.0) | 136.0 (122.0-148.0) | 138.0 (123.0-153.0) | **<0.001** |
| Platelets (10^9^/L) | 192.0 (156.0-235.0) | 183.0 (150.0-227.0) | 189.0 (154.0-232.5) | 204.0 (165.0-244.0) | **<0.001** |
| ALT (U/L) | 19.8 (13.9-30.5) | 20.1 (14.3-30.2) | 19.3 (13.4-30.4) | 19.9 (14.1-31.0) | 0.392 |
| AST (U/L) | 19.4 (15.3-27.0) | 19.5 (15.4-27.3) | 19.3 (15.1-27.0) | 19.4 (15.4-26.5) | 0.872 |
| TBil (umol/L) | 12.6 (8.8-18.0) | 13.2 (9.1-18.5) | 12.9 (9.0-18.4) | 11.7 (8.4-17.1) | **<0.001** |
| Albumin (g/L) | 40.2 (37.4-42.6) | 39.5 (36.7-42.1) | 40.2 (37.6-42.7) | 40.4 (37.7-43.2) | **<0.001** |
| BUN (mmol/L) | 6.65 (5.19-8.69) | 6.75 (5.16-8.83) | 6.57 (5.08-8.51) | 6.68 (5.36-8.61) | 0.204 |
| Creatinine (umol/L) | 85.5 (72.1-105.3) | 85.2 (72.10-103.6) | 84.5 (71.8-104.3) | 86.7 (72.7-108.4) | 0.139 |
| eGFR (ml/min/1.73m^2^) | 77.2 (58.4-91.5) | 77.4 (58.3-90.6) | 77.3 (60.0-92.1) | 76.0 (57.0-91.5) | 0.341 |
| FBG (mmol/L) | 5.50 (4.78-6.74) | 4.87 (4.41-5.55) | 5.66 (4.89-6.80) | 6.31 (5.28-8.48) | **<0.001** |
| TG (mmol/L) | 1.20 (0.86-1.73) | 0.80 (0.67-0.97) | 1.26 (1.02-1.46) | 2.01 (1.58-2.57) | **<0.001** |
| TC (mmol/L) | 3.76 (3.08-4.52) | 3.51 (2.92-4.20) | 3.73 (3.08-4.44) | 4.09 (3.36-4.97) | **<0.001** |
| LDL-C (mmol/L) | 2.28 (1.73-2.96) | 2.05 (1.59-2.69) | 2.31 (1.80-2.93) | 2.53 (1.90-3.26) | **<0.001** |
| HDL-C (mmol/L) | 0.99 (0.82-1.18) | 1.11 (0.93-1.33) | 0.99 (0.83-1.16) | 0.89 (0.76-1.03) | **<0.001** |
| Potassium (mmol/L) | 3.97 (3.67-4.28) | 3.96 (3.69-4.30) | 3.95 (3.66-4.25) | 3.98 (3.69-4.30) | 0.226 |
| Sodium (mmol/L) | 141.0 (138.7-143.0) | 141.1 (138.8-143.0) | 141.2 (138.9-143.2) | 140.7 (138.4-142.7) | **0.015** |
| LDH (U/L) | 186.1 (156.2-228.4) | 188.6 (157.2-232.4) | 185.9 (156.6-228.6) | 184.1 (154.6-225.4) | 0.269 |
| cTnT (ng/ml) | 0.024 (0.013-0.058) | 0.022 (0.012-0.058) | 0.025 (0.013- 0.060) | 0.024 (0.013-0.057) | 0.361 |
| NT-proBNP (pg/ml) | 1555.0 (660.4-3989.0) | 1652.0 (688.8-4047.5) | 1550.0 (685.1-4085.0) | 1455.0 (630.0-3730.5) | 0.173 |
| Medications at discharge (%) |  |  |  |  |  |
| Antiplatelet agents | 1788 (59.19%) | 575 (57.10%) | 627 (62.26%) | 586 (58.19%) | **0.046** |
| Statins | 1863 (61.67%) | 619 (61.47%) | 640 (63.56%) | 604 (59.98%) | 0.253 |
| Fenofibrate | 4 (0.13%) | 1 (0.10%) | 1 (0.10%) | 2 (0.20%) | 0.999 |
| Other lipid-lowering drugs | 232 (7.68%) | 82 (8.14%) | 67 (6.65%) | 83 (8.24%) | 0.325 |
| ACEI/ARB | 1354 (44.82%) | 432 (42.90%) | 470 (46.67%) | 452 (44.89%) | 0.234 |
| ARNI | 30 (0.99%) | 10 (0.99%) | 6 (0.60%) | 14 (1.39%) | 0.199 |
| β‑blocker | 2163 (71.60%) | 687 (68.22%) | 735 (72.99%) | 741 (73.58%) | **0.014** |
| CCB | 722 (23.90%) | 225 (22.34%) | 252 (25.02%) | 245 (24.33%) | 0.342 |
| Mineralocorticoid antagonists | 1904 (63.03%) | 622 (61.77%) | 644 (63.95%) | 638 (63.36%) | 0.576 |
| Diuretics | 1454 (48.13%) | 461 (45.78%) | 485 (48.16%) | 508 (50.45%) | 0.111 |
| Nitrates | 1298 (42.97%) | 422 (41.91%) | 450 (44.69%) | 426 (42.30%) | 0.395 |
| Digoxin | 1046 (34.62%) | 332 (32.97%) | 369 (36.64%) | 345 (34.26%) | 0.213 |
| Insulin | 408 (13.51%) | 138 (13.70%) | 127 (12.61%) | 143 (14.20%) | 0.566 |
| SGLT2 inhibitors | 40 (1.32%) | 12 (1.19%) | 10 (0.99%) | 18 (1.79%) | 0.268 |
| Other oral antidiabetic agents | 668 (22.11%) | 223 (22.14%) | 220 (21.85%) | 225 (22.34%) | 0.964 |

*TyG index* triglyceride-glucose index, *PSM* propensity score matching, *BMI* body mass index, *SBP* systolic blood pressure, *DBP* diastolic blood pressure, *HR* heart rate, *LVEF* left ventricular ejection fraction*, NYHA* New York Heart Association, *AF* atrial fibrillation, *CKD* chronic kidney disease, *MI* myocardial infarction, *PAD* peripheral arterial disease, *COPD* chronic obstructive pulmonary disease, *MetS* metabolic syndrome, *PCI* percutaneous coronary intervention, CABG coronary artery bypass grafting, *WBC* white blood cell, *ALT* alanine aminotransferase, *AST* aspartate aminotransferase, *TBil* total bilirubin, *BUN* blood urea nitrogen, *eGFR* estimated glomerular filtration rate, *FBG* fasting blood glucose, *TG* triglyceride, *TC* total cholesterol, *LDL-C* low-density lipoprotein cholesterol, *HDL*-*C* high-density lipoprotein cholesterol, *LDH* lactic dehydrogenase, *cTnT* cardiac troponin T, *NT*-*proBNP* N-terminal pro-brain natriuretic peptide, *ACEI/ARB* angiotensin converting enzyme inhibitor/angiotensin receptor blocker, *ARNI* angiotensin receptor-neprilysin inhibitors, *CCB* calcium channel blockers, *SGLT2 inhibitors* sodium-glucose co-transporter-2 inhibitors, *CV death* cardiovascular death. *P* values < 0.05 are presented in bold

| **Table S2 HRs of primary outcomes according to TyG index tertiles after PSM analysis.** | | | | | | | | |
| --- | --- | --- | --- | --- | --- | --- | --- | --- |
| **Categories** | **Incidence/**  **1000 person-y** | **Univariate analysis** | | |  | **Multivariate analysis** | | |
|  |  | **HR (95% CI)** | ***P*-value** | ***P* for trend** |  | **HR (95% CI)** | ***P*-value** | ***P* for trend** |
| All-cause death |  |  |  |  |  |  |  |  |
| Continuousvariable per 1 unit |  | 1.40 (1.26-1.56) | **<0.001** |  |  | 1.80 (1.57-2.06) | **<0.001** |  |
| Tertile^a^ | 63.38 |  |  | **<0.001** |  |  |  | **<0.001** |
| T1 (n=1007) | 48.06 | Ref. |  |  |  | Ref. |  |  |
| T2 (n=1007) | 62.88 | 1.31 (1.10-1.55) | **0.002** |  |  | 1.44 (1.20-1.72) | **<0.001** |  |
| T3 (n=1007) | 80.63 | 1.67 (1.42-1.97) | **<0.001** |  |  | 2.10 (1.73-2.54) | **<0.001** |  |
| CV death |  |  |  |  |  |  |  |  |
| Continuousvariable per 1 unit |  | 1.47 (1.28-1.69) | **<0.001** |  |  | 1.77 (1.49-2.12) | **<0.001** |  |
| Tertile | 38.42 |  |  | **<0.001** |  |  |  | **<0.001** |
| T1 (n=1007) | 27.20 | Ref. |  |  |  | Ref. |  |  |
| T2 (n=1007) | 38.89 | 1.43 (1.14-1.78) | **0.002** |  |  | 1.53 (1.21-1.94) | **<0.001** |  |
| T3 (n=1007) | 50.11 | 1.84 (1.48-2.27) | .. **<0.001** |  |  | 2.14 (1.67-2.75) | **<0.001** |  |

*CI* confidence interval, *HR* hazard ratio, *TyG index* triglyceride-glucose index, *PSM* propensity score matching

Multivariate analysis: adjusted for age, gender, body mass index, heart rate, smoking status, white blood cell, hemoglobin, platelets, TBil, albumin, total cholesterol, LDL-C, HDL-C, sodium, previous cardiac valve surgery, antiplatelet agents and β-blocker.

^a^TyG index: T1 (< 8.40), T2 (8.40-8.93), T3 (≥ 8.93). *P* values < 0.05 are presented in bold

| **Table S3 HRs of primary outcomes according to TyG index tertiles in different metabolic status groups** | | | | | | | | | | | | |
| --- | --- | --- | --- | --- | --- | --- | --- | --- | --- | --- | --- | --- |
| **Subgroups** | **Events (%)** | **Unadjusted** |  |  |  | **Model 1** |  |  |  | **Model 2** |  |  |
|  |  | **HR (95% CI)** | ***P*-value** | ***P* for trend** |  | **HR (95% CI)** | ***P*-value** | ***P* for trend** |  | **HR (95% CI)** | ***P*-value** | ***P* for trend** |
| MetS Group |  |  |  |  |  |  |  |  |  |  |  |  |
| All-cause death |  |  |  |  |  |  |  |  |  |  |  |  |
| Continuous variable per 1 unit |  | 1.44 (1.31-1.57) | **<0.001** |  |  | 1.77 (1.60-1.97) | **<0.001** |  |  | 1.67 (1.50-1.87) | **<0.001** |  |
| Tertile^a^ | 1170 (35.8) |  |  | **<0.001** |  |  |  | **<0.001** |  |  |  | **<0.001** |
| T1 (n=1089) | 278 (25.5) | Ref. |  |  |  | Ref. |  |  |  | Ref. |  |  |
| T2 (n=1089) | 422 (38.8) | 1.67 (1.44-1.94) | **<0.001** |  |  | 1.88 (1.61-2.20) | **<0.001** |  |  | 1.90 (1.62-2.22) | **<0.001** |  |
| T3 (n=1089) | 470 (43.2) | 1.94 (1.67-2.25) | **<0.001** |  |  | 2.43 (2.07-2.86) | **<0.001** |  |  | 2.26 (1.91-2.66) | **<0.001** |  |
| CV death |  |  |  |  |  |  |  |  |  |  |  |  |
| Continuous variable per 1 unit |  | 1.44 (1.29-1.61) | **<0.001** |  |  | 1.74 (1.53-1.99) | **<0.001** |  |  | 1.66 (1.44-1.90) | **<0.001** |  |
| Tertile^a^ | 727 (22.3) |  |  | **<0.001** |  |  |  | **<0.001** |  |  |  | **<0.001** |
| T1 (n=1089) | 176 (16.2) | Ref. |  |  |  | Ref. |  |  |  | Ref. |  |  |
| T2 (n=1089) | 253 (23.2) | 1.57 (1.30-1.91) | **<0.001** |  |  | 1.81 (1.48-2.21) | **<0.001** |  |  | 1.81 (1.48-2.21) | **<0.001** |  |
| T3 (n=1089) | 298 (27.4) | 1.93 (1.60-2.33) | **<0.001** |  |  | 2.41 (1.96-2.95) | **<0.001** |  |  | 2.26 (1.84-2.79) | **<0.001** |  |
| Non-MetS Group |  |  |  |  |  |  |  |  |  |  |  |  |
| All-cause death |  |  |  |  |  |  |  |  |  |  |  |  |
| Continuous variable per 1 unit |  | 1.22 (1.07-1.39) | **..0.003** |  |  | 1.50 (1.30-1.73) | **<0.001** |  |  | 1.40 (1.19-1.64) | **<0.001** |  |
| Tertile^b^ | 988 (28.8) |  |  | **...0.009** |  |  |  | **<0.001** |  |  |  | **<0.001** |
| T1 (n=1143) | 316 (27.6) | Ref. |  |  |  | Ref. |  |  |  | Ref. |  |  |
| T2 (n=1143) | 312 (27.3) | 1.00 (0.85-1.17) | ...0.992 |  |  | 1.18 (1.00-1.39) | **...0.044** |  |  | 1.21 (1.02-1.42) | **...0.027** |  |
| T3 (n=1144) | 360 (31.5) | 1.23 (1.05-1.43) | **..0.008** |  |  | 1.52 (1.29-1.80) | **<0.001** |  |  | 1.41 (1.18-1.68) | **<0.001** |  |
| CV death |  |  |  |  |  |  |  |  |  |  |  |  |
| Continuous variable per 1 unit |  | 1.31 (1.11-1.55) | **..0.002** |  |  | 1.56 (1.29-1.87) | **<0.001** |  |  | 1.46 (1.19-1.80) | **<0.001** |  |
| Tertile^b^ | 578 (16.9) |  |  | **<0.001** |  |  |  | **<0.001** |  |  |  | **<0.001** |
| **Table S3 (continued)** | | | | | | | | | | | | |
| **Subgroups** | **Events (%)** | **Unadjusted** |  |  |  | **Model 1** |  |  |  | **Model 2** |  |  |
|  |  | **HR (95% CI)** | ***P*-value** | ***P* for trend** |  | **HR (95% CI)** | ***P*-value** | ***P* for trend** |  | **HR (95% CI)** | ***P*-value** | ***P* for trend** |
| T1 (n=1143) | 166 (14.5) | Ref. |  |  |  | Ref. |  |  |  | Ref. |  |  |
| T2 (n=1143) | 195 (17.1) | 1.19 (0.97-1.47) | ...0.096 |  |  | 1.40 (1.13-1.74) | **..0.002** |  |  | 1.47 (1.18-1.83) | **<0.001** |  |
| T3 (n=1144) | 217 (19.0) | 1.41 (1.15-1.73) | **<0.001** |  |  | 1.72 (1.38-2.15) | **<0.001** |  |  | 1.61 (1.27-2.04) | **<0.001** |  |

*CI* confidence interval, *HR* hazard ratio, *TyG index* triglyceride–glucose index, *MetS* metabolic syndrome

Model 1: adjusted for age, gender, body mass index, smoking status, drinking status, hemoglobin, ALT, AST, TBil, albumin, eGFR, total cholesterol, LDL-C, HDL-C, cTnT, sodium, LVEF, NT-proBNP and NYHA classification.

Model 2: adjusted for Model 1 + hypertension, diabetes, atrial fibrillation, previous MI, angina, stroke, COPD, previous heart surgery, antiplatelet agent, lipid-lowering drugs, ACEI/ARB, ARNI, β-blocker, mineralocorticoid antagonist, diuretics, digoxin and hypoglycemic therapy.

^a^TyG index: T1 (< 8.80), T2 (8.80-9.25), T3 (≥ 9.25); ^b^TyG index: T1 (< 8.14), T2 (8.14-8.56), T3 (≥ 8.56). *P* values < 0.05 are presented in bold

| **Table S4 HRs of primary outcomes according to TyG index tertiles in different heart failure phenotypes** | | | | | | | | | | | | |
| --- | --- | --- | --- | --- | --- | --- | --- | --- | --- | --- | --- | --- |
| **Subgroups** | **Events (%)** | **Unadjusted** |  |  |  | **Model 1** |  |  |  | **Model 2** |  |  |
|  |  | **HR (95% CI)** | ***P*-value** | ***P* for trend** |  | **HR (95% CI)** | ***P*-value** | ***P* for trend** |  | **HR (95% CI)** | ***P*-value** | ***P* for trend** |
| HFrEF Group |  |  |  |  |  |  |  |  |  |  |  |  |
| All-cause death |  |  |  |  |  |  |  |  |  |  |  |  |
| Continuous variable per 1 unit |  | 1.06 (0.95-1.19) | ...0.313 |  |  | 1.22 (1.06-1.39) | **..0.004** |  |  | 1.07 (0.92-1.25) | ...0.403 |  |
| Tertile^a^ | 776 (34.9) |  |  | ...0.262 |  |  |  | **..0.002** |  |  |  | ...0.170 |
| T1 (n=740) | 258 (34.9) | Ref. |  |  |  | Ref. |  |  |  | Ref. |  |  |
| T2 (n=740) | 252 (34.1) | 1.00 (0.84-1.19) | ...0.996 |  |  | 1.11 (0.92-1.33) | ...0.275 |  |  | 1.05 (0.87-1.27) | ...0.616 |  |
| T3 (n=741) | 266 (35.9) | 1.10 (0.93-1.31) | ...0.267 |  |  | 1.37 (1.13-1.67) | **..0.002** |  |  | 1.16 (0.94-1.45) | ...0.169 |  |
| CV death |  |  |  |  |  |  |  |  |  |  |  |  |
| Continuous variable per 1 unit |  | 1.07 (0.94-1.23) | ...0.299 |  |  | 1.22 (1.04-1.44) | **..0.014** |  |  | 1.08 (0.89-1.30) | ...0.430 |  |
| Tertile^a^ | 542 (24.4) |  |  | ...0.156 |  |  |  | **..0.003** |  |  |  | ...0.142 |
| T1 (n=740) | 173 (23.4) | Ref. |  |  |  | Ref. |  |  |  | Ref. |  |  |
| T2 (n=740) | 182 (24.6) | 1.08 (0.88-1.33) | ...0.464 |  |  | 1.18 (0.95-1.47) | ...0.131 |  |  | 1.10 (0.88-1.38) | ...0.400 |  |
| T3 (n=741) | 187 (25.2) | 1.16 (0.94-1.43) | ...0.156 |  |  | 1.44 (1.14-1.82) | **..0.003** |  |  | 1.22 (0.94-1.58) | ...0.142 |  |
| HFmrEF Group |  |  |  |  |  |  |  |  |  |  |  |  |
| All-cause death |  |  |  |  |  |  |  |  |  |  |  |  |
| Continuous variable per 1 unit |  | 1.35 (1.18-1.55) | **<0.001** |  |  | 1.74 (1.44-2.09) | **<0.001** |  |  | 1.52 (1.23-1.87) | **<0.001** |  |
| Tertile^b^ | 436 (30.4) |  |  | **<0.001** |  |  |  | **<0.001** |  |  |  | **..0.048** |
| T1 (n=478) | 122 (25.5) | Ref. |  |  |  | Ref. |  |  |  | Ref. |  |  |
| T2 (n=477) | 148 (31.0) | 1.22 (0.96-1.54) | ...0.112 |  |  | 1.36 (1.06-1.75) | **..0.017** |  |  | 1.27 (0.98-1.65) | ....0.071 |  |
| T3 (n=478) | 166 (34.7) | 1.49 (1.18-1.88) | **<0.001** |  |  | 1.64 (1.25-2.15) | **<0.001** |  |  | 1.36 (1.01-1.81) | **..0.041** |  |
| CV death |  |  |  |  |  |  |  |  |  |  |  |  |
| Continuous variable per 1 unit |  | 1.52 (1.27-1.82) | **<0.001** |  |  | 1.87 (1.46-2.39) | **<0.001** |  |  | 1.59 (1.20-2.10) | **0.001** |  |
| Tertile^b^ | 235 (16.4) |  |  | **<0.001** |  |  |  | **<0.001** |  |  |  | **..0.022** |
| **Table S4 (continued)** | | | | | | | | | | | | |
| **Subgroups** | **Events (%)** | **Unadjusted** |  |  |  | **Model 1** |  |  |  | **Model 2** |  |  |
|  |  | **HR (95% CI)** | ***P*-value** | ***P* for trend** |  | **HR (95% CI)** | ***P*-value** | ***P* for trend** |  | **HR (95% CI)** | ***P*-value** | ***P* for trend** |
| T1 (n=478) | 59 (12.3) | Ref. |  |  |  | Ref. |  |  |  | Ref. |  |  |
| T2 (n=477) | 76 (15.9) | 1.29 (0.92-1.82) | ...0.139 |  |  | 1.40 (0.98-1.99) | ..0.065 |  |  | 1.38 (0.95-2.00) | ...0.091 |  |
| T3 (n=478) | 100 (20.9) | 1.85 (1.34-2.55) | **<0.001** |  |  | 1.92 (1.32-2.79) | **<0.001** |  |  | 1.62 (1.08-2.43) | **. .0.019** |  |
| HFpEF Group |  |  |  |  |  |  |  |  |  |  |  |  |
| All-cause death |  |  |  |  |  |  |  |  |  |  |  |  |
| Continuous variable per 1 unit |  | 1.76 (1.61-1.93) | **<0.001** |  |  | 1.98 (1.77-2.22) | **<0.001** |  |  | 1.94 (1.71-2.21) | **<0.001** |  |
| Tertile^c^ | 946 (31.1) |  |  | **<0.001** |  |  |  | **<0.001** |  |  |  | **<0.001** |
| T1 (n=1014) | 182 (17.9) | Ref. |  |  |  | Ref. |  |  |  | Ref. |  |  |
| T2 (n=1014) | 306 (30.2) | 1.78 (1.48-2.14) | **<0.001** |  |  | 1.83 (1.51-2.22) | **<0.001** |  |  | 1.68 (1.38-2.05) | **<0.001** |  |
| T3 (n=1015) | 458 (45.1) | 3.07 (2.59-3.65) | **<0.001** |  |  | 3.36 (2.74-4.11) | **<0.001** |  |  | 3.07 (2.47-3.81) | **<0.001** |  |
| CV death |  |  |  |  |  |  |  |  |  |  |  |  |
| Continuous variable per 1 unit |  | 1.96 (1.74-2.20) | **<0.001** |  |  | 2.14 (1.84-2.49) | **<0.001** |  |  | 2.12 (1.79-2.51) | **<0.001** |  |
| Tertile^c^ | 528 (17.4) |  |  | **<0.001** |  |  |  | **<0.001** |  |  |  | **<0.001** |
| T1 (n=1014) | 84 (8.3) | Ref. |  |  |  | Ref. |  |  |  | Ref. |  |  |
| T2 (n=1014) | 165 (16.3) | 2.07 (1.59-2.69) | **<0.001** |  |  | 2.11 (1.60-2.78) | **<0.001** |  |  | 1.95 (1.47-2.60) | **<0.001** |  |
| T3 (n=1015) | 279 (27.5) | 4.03 (3.16-5.15) | **<0.001** |  |  | 4.25 (3.21-5.65) | **<0.001** |  |  | 3.93 (2.91-5.32) | **<0.001** |  |

*CI* confidence interval, *HR* hazard ratio, *TyG index* triglyceride–glucose index, *HFrEF* heart failure with reduced ejection fraction, *HFmrEF* heart failure with mildly reduced ejection fraction, *HFpEF* heart failure with preserved ejection fraction. *P* values < 0.05 are presented in bold

Model 1: adjusted for age, gender, body mass index, smoking status, drinking status, hemoglobin, ALT, AST, TBil, albumin, eGFR, total cholesterol, LDL-C, HDL-C, cTnT, sodium, LVEF, NT-proBNP and NYHA classification.

Model 2: adjusted for Model 1 + hypertension, diabetes, atrial fibrillation, previous MI, angina, stroke, COPD, previous heart surgery, antiplatelet agent, lipid-lowering drugs, ACEI/ARB, ARNI, β-blocker, mineralocorticoid antagonist, diuretics, digoxin and hypoglycemic therapy.

^a^TyG index: T1 (< 8.33), T2 (8.33-8.83), T3 (≥ 8.83); ^b^TyG index: T1 (< 8.43), T2 (8.43-8.94), T3 (≥ 8.94); ^c^TyG index: T1 (< 8.44), T2 (8.44-8.98), T3 (≥ 8.98)

| **Table S5** **HRs of primary outcomes according to TyG index tertiles among other different subgroups** | | | | | | | | | | |
| --- | --- | --- | --- | --- | --- | --- | --- | --- | --- | --- |
| **Subgroups** | **Incidence/**  **1000 person-y** | **Model 1** | | |  | **Model 2** | | |  | ***P* for**  **interaction** |
|  |  | **HR (95% CI)** | ***P*-value** | ***P* for trend** |  | **HR (95% CI)** | ***P*-value** | ***P* for trend** |  |  |
| Diabetes group |  |  |  |  |  |  |  |  |  |  |
| All-cause death |  |  |  | **<0.001** |  |  |  | **<0.001** |  | 0.686^a^ |
| T1 (n=996) | 74.65 | Ref. |  |  |  | Ref. |  |  |  |  |
| T2 (n=995) | 95.61 | 1.32 (1.14-1.52) | **<0.001** |  |  | 1.45 (1.25-1.68) | **<0.001** |  |  |  |
| T3 (n=996) | 114.65 | 1.68 (1.46-1.93) | **<0.001** |  |  | 1.95 (1.67-2.29) | **<0.001** |  |  |  |
| CV death |  |  |  | **<0.001** |  |  |  | **<0.001** |  | 0.484^a^ |
| T1 (n=996) | 43.29 | Ref. |  |  |  | Ref. |  |  |  |  |
| T2 (n=995) | 57.73 | 1.37 (1.14-1.65) | .. **<0.001** |  |  | 1.51 (1.24-1.83) | . **<0.001** |  |  |  |
| T3 (n=996) | 73.45 | 1.82 (1.52-2.18) | .. **<0.001** |  |  | 2.13 (1.74-2.60) | . **<0.001** |  |  |  |
| Non-diabetes group |  |  |  |  |  |  |  |  |  |  |
| All-cause death |  |  |  | **0.005** |  |  |  | **<0.001** |  |  |
| T1 (n=1237) | 45.64 | Ref. |  |  |  | Ref. |  |  |  |  |
| T2 (n=1236) | 48.94 | 1.08 (0.92-1.26) | 0.376 |  |  | 1.23 (1.04-1.45) | **0.018** |  |  |  |
| T3 (n=1237) | 55.57 | 1.25 (1.07-1.47) | **0.005** |  |  | 1.54 (1.27-1.86) | **<0.001** |  |  |  |
| CV death |  |  |  | **0.004** |  |  |  | **<0.001** |  |  |
| T1 (n=1237) | 26.35 | Ref. |  |  |  | Ref. |  |  |  |  |
| T2 (n=1236) | 31.05 | 1.23 (1.00-1.52) | **0.047** |  |  | 1.43 (1.15-1.79) | **0.001** |  |  |  |
| T3 (n=1237) | 32.08 | 1.36 (1.10-1.67) | **0.004** |  |  | 1.66 (1.29-2.13) | **. <0.001** |  |  |  |
| HTN group |  |  |  |  |  |  |  |  |  |  |
| All-cause death |  |  |  | **<0.001** |  |  |  | **<0.001** |  | 0.059^b^ |
| T1 (n=1381) | 51.39 | Ref. |  |  |  | Ref. |  |  |  |  |
| T2 (n=1380) | 69.38 | 1.38 (1.20-1.59) | **<0.001** |  |  | 1.35 (1.16-1.57) | **<0.001** |  |  |  |
| T3 (n=1381) | 101.73 | 2.14 (1.87-2.44) | **<0.001** |  |  | 2.01 (1.70-2.37) | **<0.001** |  |  |  |
| CV death |  |  |  | **<0.001** |  |  |  | **<0.001** |  | 0.055^b^ |
| T1 (n=1381) | 28.85 | Ref. |  |  |  | Ref. |  |  |  |  |
| T2 (n=1380) | 40.21 | 1.42 (1.18-1.71) | **<0.001** |  |  | 1.33 (1.09-1.63) | **0.005** |  |  |  |
| T3 (n=1381) | 63.66 | 2.36 (1.98-2.81) | **<0.001** |  |  | 2.05 (1.66-2.54) | **<0.001** |  |  |  |
| Non-HTN group |  |  |  |  |  |  |  |  |  |  |
| All-cause death |  |  |  | **<0.001** |  |  |  | **<0.001** |  |  |
| T1 (n=852) | 48.56 | Ref. |  |  |  | Ref. |  |  |  |  |
| T2 (n=851) | 59.88 | 1.21 (1.01-1.45) | **0.034** |  |  | 1.37 (1.08-1.58) | **0.006** |  |  |  |
| T3 (n=852) | 75.82 | 1.51 (1.26-1.80) | **<0.001** |  |  | 1.68 (1.38-1.98) | **<0.001** |  |  |  |
| CV death |  |  |  | **<0.001** |  |  |  | **<0.001** |  |  |
| T1 (n=852) | 29.81 | Ref. |  |  |  | Ref. |  |  |  |  |
| T2 (n=851) | 38.02 | 1.32 (1.05-1.66) | **0.020** |  |  | 1.45 (1.14-1.86) | **0.003** |  |  |  |
| T3 (n=852) | 46.84 | 1.64 (1.31-2.06) | **<0.001** |  |  | 1.71 (1.29-2.27) | **<0.001** |  |  |  |
| Obesity group |  |  |  |  |  |  |  |  |  |  |
| All-cause death |  |  |  | **<0.001** |  |  |  | **<0.001** |  | 0.678^c^ |
| T1 (n=539) | 38.81 | Ref. |  |  |  | Ref. |  |  |  |  |
| T2 (n=539) | 79.00 | 2.02 (1.59-2.56) | **<0.001** |  |  | 2.47 (1.90-3.21) | **<0.001** |  |  |  |
| **Table S5 (continued)** | | | | | | | | | | |
| **Subgroups** |  | **Model 1** | | **Model 2** | | | | |  | ***P* for interaction** |
|  | **Incidence/**  **1000 person-y** | **HR (95% CI)** | ***P*-value** | ***P* for trend** |  | **HR (95% CI)** | ***P*-value** | ***P* for trend** |  |  |
| T3 (n=539) | 85.82 | 2.30 (1.82-2.91) | **<0.001** |  |  | 3.13 (2.36-4.16) | **<0.001** |  |  |  |
| CV death |  |  |  | **<0.001** |  |  |  | **<0.001** |  | 0.924^c^ |
| T1 (n=539) | 23.14 | Ref. |  |  |  | Ref. |  |  |  |  |
| T2 (n=539) | 48.89 | 2.10 (1.55-2.85) | **<0.001** |  |  | 2.47 (1.77-3.46) | **<0.001** |  |  |  |
| T3 (n=539) | 52.22 | 2.33 (1.72-3.15) | **<0.001** |  |  | 2.96 (2.06-4.26) | **<0.001** |  |  |  |
| Non-obesity group |  |  |  |  |  |  |  |  |  |  |
| All-cause death |  |  |  | **<0.001** |  |  |  | **<0.001** |  |  |
| T1 (n=1693) | 52.70 | Ref. |  |  |  | Ref. |  |  |  |  |
| T2 (n=1693) | 63.09 | 1.19 (1.05-1.35) | **0.007** |  |  | 1.20 (1.05-1.37) | **0.009** |  |  |  |
| T3 (n=1694) | 93.36 | 1.74 (1.54-1.96) | **<0.001** |  |  | 1.69 (1.46-1.97) | **<0.001** |  |  |  |
| CV death |  |  |  | **<0.001** |  |  |  | **<0.001** |  |  |
| T1 (n=1693) | 29.71 | Ref. |  |  |  | Ref. |  |  |  |  |
| T2 (n=1693) | 38.17 | 1.28 (1.08-1.51) | **0.004** |  |  | 1.27 (1.06-1.52) | **0.008** |  |  |  |
| T3 (n=1694) | 58.57 | 1.93 (1.65-2.26) | **<0.001** |  |  | 1.91 (1.57-2.33) | **<0.001** |  |  |  |
| Dyslipidemia group |  |  |  |  |  |  |  |  |  |  |
| All-cause death |  |  |  | **<0.001** |  |  |  | **<0.001** |  | 0.977^d^ |
| T1 (n=1395) | 48.89 | Ref. |  |  |  | Ref. |  |  |  |  |
| T2 (n=1395) | 68.56 | 1.31 (1.13-1.50) | **<0.001** |  |  | 1.45 (1.25-1.69) | **<0.001** |  |  |  |
| T3 (n=1396) | 92.79 | 1.78 (1.56-2.04) | **<0.001** |  |  | 1.95 (1.67-2.29) | **<0.001** |  |  |  |
| CV death |  |  |  | **<0.001** |  |  |  | **<0.001** |  | 0.779^d^ |
| T1 (n=1395) | 30.91 | Ref. |  |  |  | Ref. |  |  |  |  |
| T2 (n=1395) | 42.91 | 1.30 (1.08-1.55) | **0.004** |  |  | 1.45 (1.20-1.75) | **<0.001** |  |  |  |
| T3 (n=1396) | 59.44 | 1.80 (1.52-2.13) | **<0.001** |  |  | 1.97 (1.61-2.40) | **<0.001** |  |  |  |
| Non-dyslipidemia group |  |  |  |  |  |  |  |  |  |  |
| All-cause death |  |  |  | **<0.001** |  |  |  | **<0.001** |  |  |
| T1 (n=837) | 51.80 | Ref. |  |  |  | Ref. |  |  |  |  |
| T2 (n=837) | 60.71 | 1.20 (1.00-1.44) | **0.050** |  |  | 1.31 (1.08-1.58) | **0.007** |  |  |  |
| T3 (n=837) | 90.55 | 1.78 (1.50-2.11) | **<0.001** |  |  | 1.76 (1.43-2.17) | **<0.001** |  |  |  |
| CV death |  |  |  | **<0.001** |  |  |  | **<0.001** |  |  |
| T1 (n=837) | 27.57 | Ref. |  |  |  | Ref. |  |  |  |  |
| T2 (n=837) | 36.03 | 1.34 (1.05-1.70) | **0.020** |  |  | 1.41 (1.09-1.83) | **0.009** |  |  |  |
| T3 (n=837) | 49.37 | 1.81 (1.44-2.28) | **<0.001** |  |  | 1.78 (1.35-2.36) | **<0.001** |  |  |  |
| Ischemic etiology |  |  |  |  |  |  |  |  |  |  |
| All-cause death |  |  |  | **<0.001** |  |  |  | **<0.001** |  | 0.052^e^ |
| T1 (n=1059) | 58.27 | Ref. |  |  |  | Ref. |  |  |  |  |
| T2 (n=1059) | 70.46 | 1.27 (1.09-1.48) | **0.002** |  |  | 1.27 (1.08-1.50) | **0.005** |  |  |  |
| T3 (n=1059) | 97.73 | 1.82 (1.58-2.11) | **<0.001** |  |  | 1.68 (1.40-2.03) | **<0.001** |  |  |  |
| CV death |  |  |  | **<0.001** |  |  |  | **<0.001** |  | 0.285^e^ |
| T1 (n=1059) | 33.11 | Ref. |  |  |  | Ref. |  |  |  |  |
| **Table S5 (continued)** | | | | | | | | | | |
| **Subgroups** | **Incidence/**  **1000 person-y** | **Model 1** | | |  | **Model 2** | | |  | ***P* for interaction** |
|  |  | **HR (95% CI)** | ***P*-value** | ***P* for trend** |  | **HR (95% CI)** | ***P*-value** | ***P* for trend** |  |  |
| T2 (n=1059) | 43.27 | 1.37 (1.12-1.67) | **0.002** |  |  | 1.33 (1.08-1.65) | **0.009** |  |  |  |
| T3 (n=1059) | 63.11 | 2.03 (1.69-2.45) | **<0.001** |  |  | 1.87 (1.47-2.36) | **<0.001** |  |  |  |
| Non-ischemic etiology |  |  |  |  |  |  |  |  |  |  |
| All-cause death |  |  |  | **<0.001** |  |  |  | **<0.001** |  |  |
| T1 (n=1173) | 43.47 | Ref. |  |  |  | Ref. |  |  |  |  |
| T2 (n=1173) | 60.41 | 1.41 (1.19-1.66) | **<0.001** |  |  | 1.46 (1.22-1.73) | **<0.001** |  |  |  |
| T3 (n=1174) | 86.40 | 2.06 (1.76-2.40) | **<0.001** |  |  | 2.26 (1.87-2.74) | **<0.001** |  |  |  |
| CV death |  |  |  | **<0.001** |  |  |  | **<0.001** |  |  |
| T1 (n=1173) | 23.86 | Ref. |  |  |  | Ref. |  |  |  |  |
| T2 (n=1173) | 38.76 | 1.64 (1.33-2.03) | **<0.001** |  |  | 1.72 (1.37-2.16) | **<0.001** |  |  |  |
| T3 (n=1174) | 50.40 | 2.18 (1.77-2.68) | **<0.001** |  |  | 2.48 (1.93-3.19) | **<0.001** |  |  |  |

*CI* confidence interval, *HR* hazard ratio, *TyG index* triglyceride–glucose index, *HTN* hypertension

Model 1: adjusted for age and gender.

Model 2: adjusted for Model 1 + body mass index, smoking status, drinking status, hemoglobin, ALT, AST, TBil, albumin, eGFR, total cholesterol, LDL-C, HDL-C, cTnT, sodium, LVEF, NT-proBNP, NYHA classification, hypertension, diabetes, atrial fibrillation, previous MI, angina, stroke, COPD, previous heart surgery, antiplatelet agents, lipid-lowering drugs, ACEI/ARB, ARNI, β-blocker, mineralocorticoid antagonist, diuretics, digoxin and hypoglycemic therapy (except for variables used for grouping).

According to the diagnostic criteria of metabolic syndrome in this study, the obesity was defined by BMI ≥ 28 kg/m^2^ and the dyslipidemia was defined by fasting TG ≥ 1.7 mmol/L or fasting HDL-C < 1.04 mmol/L. Ischemic etiology included the previous myocardial infarction, angina, the history of PCI or CABG surgery. *P* values < 0.05 are presented in bold

^a^*P* for interaction between the TyG index and the diabetes with all-cause death and CV death as endpoint, respectively.

^b^*P* for interaction between the TyG index and the hypertension with all-cause death and CV death as endpoint, respectively.

^c^*P* for interaction between the TyG index and the obesity with all-cause death and CV death as endpoint, respectively.

^d^*P* for interaction between the TyG index and the dyslipidemia with all-cause death and CV death as endpoint, respectively.

^e^*P* for interaction between the TyG index and the ischemic etiology with all-cause death and CV death as endpoint, respectively.
