# Supplementary material for: Correction to: Association between the triglyceride-glucose index and the risk of mortality among patients with chronic heart failure: results from a retrospective cohort study in China
Source: Cardiovasc Diabetol. 2023 Sep 15;22:250. doi: 10.1186/s12933-023-01978-2 (PMC10504743; doi:10.1186/s12933-023-01978-2)
Supplement: Supplementary file 1 — Supplementary Material 1 [file 12933_2023_1978_MOESM1_ESM.docx]

| **Table S1 Baseline characteristics of the study population according to TyG index tertiles after PSM analysis** | | | | | |
| --- | --- | --- | --- | --- | --- |
| **Variables** | **Total** | **Tertile of TyG index** | | | ***P* value** |
|  |  | **T1** | **T2** | **T3** |  |
|  | **n=3021** | **n=1007** | **n=1007** | **n=1007** |  |
| TyG index | 8.68 ± 0.59 | < 8.40 | 8.40-8.93 | ≥ 8.93 | - |
| Demographics |  |  |  |  |  |
| Age (years) | 64.2 (54.1-73.8) | 64.8 (54.5-73.8) | 64.4 (55.0-73.8) | 63.7 (53.2-74.0) | 0.632 |
| Male (%) | 2104 (69.65%) | 685 (68.02%) | 725 (72.00%) | 694 (68.92%) | 0.126 |
| BMI (kg/m^2^) | 25.3 (22.9-28.1) | 24.4 (22.1-27.2) | 25.4 (22.9-28.1) | 26.1 (23.5-28.6) | **<0.001** |
| Medical measurements |  |  |  |  |  |
| SBP (mmHg) | 130.0 (116.0-145.0) | 130.0 (116.0-145.0) | 129.0 (116.0-144.0) | 129.0 (116.0-144.0) | 0.880 |
| DBP (mmHg) | 75.0 (66.0-84.0) | 74.0 (65.0-84.0) | 75.0 (67.0-85.0) | 76.0 (66.0-85.0) | 0.059 |
| HR (bpm) | 78.0 (70.0-90.0) | 77.0 (69.0-90.0) | 79.0 (70.0-90.0) | 80.0 (70.0-92.0) | **0.005** |
| Smoking (%) |  |  |  |  | **0.014** |
| Current smoker | 760 (25.16%) | 217 (21.55%) | 267 (26.51%) | 276 (27.41%) |  |
| Former smoker | 613 (20.29%) | 214 (21.25%) | 189 (18.77%) | 210 (20.85%) |  |
| Never smoker | 1648 (54.55%) | 576 (57.20%) | 551 (54.72) | 521 (51.74%) |  |
| Drinking (%) |  |  |  |  | 0.149 |
| Current drinker | 734 (24.30%) | 229 (22.74%) | 242 (24.03%) | 263 (26.12%) |  |
| Former drinker | 300 (9.93%) | 104 (10.33%) | 87 (8.64%) | 109 (10.82%) |  |
| Never drinker | 1987 (65.77%) | 674 (66.93%) | 678 (67.33%) | 635 (63.06%) |  |
| LVEF (%) |  |  |  |  | 0.116 |
| ≤ 40% | 941 (31.15%) | 314 (31.18%) | 336 (33.37%) | 291 (28.90%) |  |
| 41%~49% | 681 (22.54%) | 233 (23.14%) | 230 (22.84%) | 218 (21.65%) |  |
| ≥ 50% | 1399 (46.31%) | 460 (45.68%) | 441 (43.79%) | 498 (49.45%) |  |
| NYHA classification (%) |  |  |  |  | 0.818 |
| I-II | 1352 (44.75%) | 445 (44.19%) | 446 (44.29%) | 461 (45.78%) |  |
| III | 1296 (42.90%) | 443 (43.99%) | 429 (42.60%) | 424 (42.11%) |  |
| IV | 373 (12.35%) | 119 (11.82%) | 132 (13.11%) | 122 (12.12%) |  |
| Medical history (%) |  |  |  |  |  |
| Hypertension | 1996 (66.07%) | 666 (66.14%) | 673 (66.83%) | 657 (65.24%) | 0.752 |
| Diabetes | 1465 (48.49%) | 485 (48.16%) | 491 (48.76%) | 489 (48.56%) | 0.964 |
| AF | 934 (30.92%) | 328 (32.57%) | 312 (30.98%) | 294 (29.20%) | 0.261 |
| CKD (Stages III-IV) | 801 (26.51%) | 269 (26.71%) | 252 (25.02%) | 280 (27.81%) | 0.363 |
| Previous MI | 1065 (35.25%) | 351 (34.86%) | 382 (37.93%) | 332 (32.97%) | 0.063 |
| Angina | 942 (31.18%) | 305 (30.29%) | 327 (32.47%) | 310 (30.78%) | 0.540 |
| Stroke | 583 (19.30%) | 199 (19.76%) | 193 (19.17%) | 191 (18.97%) | 0.895 |
| PAD | 468 (15.49%) | 165 (16.39%) | 145 (14.40%) | 158 (15.69%) | 0.458 |
| COPD | 120 (3.97%) | 31 (3.08%) | 40 (3.97%) | 49 (4.87%) | 0.121 |
| Previous heart surgery (%) |  |  |  |  |  |
| PCI | 624 (20.66%) | 212 (21.05%) | 224 (22.24%) | 188 (18.67%) | 0.131 |
| CABG | 136 (4.50%) | 54 (5.36%) | 48 (4.77%) | 34 (3.38%) | 0.088 |
| Cardiac valve surgery | 101 (3.34%) | 37 (3.67%) | 42 (4.17%) | 22 (2.18%) | **0.036** |
| Pacemaker therapy | 126 (4.17%) | 38 (3.77%) | 41 (4.07%) | 47 (4.67%) | 0.594 |
| **Table S1 (continued)** |  |  |  |  |  |
| **Variables** | **Total** | **T1** | **T2** | **T3** | ***P* value** |
| Laboratory measurements |  |  |  |  |  |
| WBC (10^9^/L) | 6.61 (5.40-8.11) | 6.14 (5.09-7.49) | 6.64 (5.38-8.03) | 7.10 (5.96-8.63) | **<0.001** |
| Hemoglobin (g/L) | 136.0 (121.0-149.0) | 134.0 (117.0-146.0) | 136.0 (122.0-148.0) | 138.0 (123.0-153.0) | **<0.001** |
| Platelets (10^9^/L) | 192.0 (156.0-235.0) | 183.0 (150.0-227.0) | 189.0 (154.0-232.5) | 204.0 (165.0-244.0) | **<0.001** |
| ALT (U/L) | 19.8 (13.9-30.5) | 20.1 (14.3-30.2) | 19.3 (13.4-30.4) | 19.9 (14.1-31.0) | 0.392 |
| AST (U/L) | 19.4 (15.3-27.0) | 19.5 (15.4-27.3) | 19.3 (15.1-27.0) | 19.4 (15.4-26.5) | 0.872 |
| TBil (umol/L) | 12.6 (8.8-18.0) | 13.2 (9.1-18.5) | 12.9 (9.0-18.4) | 11.7 (8.4-17.1) | **<0.001** |
| Albumin (g/L) | 40.2 (37.4-42.6) | 39.5 (36.7-42.1) | 40.2 (37.6-42.7) | 40.4 (37.7-43.2) | **<0.001** |
| BUN (mmol/L) | 6.65 (5.19-8.69) | 6.75 (5.16-8.83) | 6.57 (5.08-8.51) | 6.68 (5.36-8.61) | 0.204 |
| Creatinine (umol/L) | 85.5 (72.1-105.3) | 85.2 (72.10-103.6) | 84.5 (71.8-104.3) | 86.7 (72.7-108.4) | 0.139 |
| eGFR (ml/min/1.73m^2^) | 77.2 (58.4-91.5) | 77.4 (58.3-90.6) | 77.3 (60.0-92.1) | 76.0 (57.0-91.5) | 0.341 |
| FBG (mmol/L) | 5.50 (4.78-6.74) | 4.87 (4.41-5.55) | 5.66 (4.89-6.80) | 6.31 (5.28-8.48) | **<0.001** |
| TG (mmol/L) | 1.20 (0.86-1.73) | 0.80 (0.67-0.97) | 1.26 (1.02-1.46) | 2.01 (1.58-2.57) | **<0.001** |
| TC (mmol/L) | 3.76 (3.08-4.52) | 3.51 (2.92-4.20) | 3.73 (3.08-4.44) | 4.09 (3.36-4.97) | **<0.001** |
| LDL-C (mmol/L) | 2.28 (1.73-2.96) | 2.05 (1.59-2.69) | 2.31 (1.80-2.93) | 2.53 (1.90-3.26) | **<0.001** |
| HDL-C (mmol/L) | 0.99 (0.82-1.18) | 1.11 (0.93-1.33) | 0.99 (0.83-1.16) | 0.89 (0.76-1.03) | **<0.001** |
| Potassium (mmol/L) | 3.97 (3.67-4.28) | 3.96 (3.69-4.30) | 3.95 (3.66-4.25) | 3.98 (3.69-4.30) | 0.226 |
| Sodium (mmol/L) | 141.0 (138.7-143.0) | 141.1 (138.8-143.0) | 141.2 (138.9-143.2) | 140.7 (138.4-142.7) | **0.015** |
| LDH (U/L) | 186.1 (156.2-228.4) | 188.6 (157.2-232.4) | 185.9 (156.6-228.6) | 184.1 (154.6-225.4) | 0.269 |
| cTnT (ng/ml) | 0.024 (0.013-0.058) | 0.022 (0.012-0.058) | 0.025 (0.013- 0.060) | 0.024 (0.013-0.057) | 0.361 |
| NT-proBNP (pg/ml) | 1555.0 (660.4-3989.0) | 1652.0 (688.8-4047.5) | 1550.0 (685.1-4085.0) | 1455.0 (630.0-3730.5) | 0.173 |
| Medications at discharge (%) |  |  |  |  |  |
| Antiplatelet agents | 1788 (59.19%) | 575 (57.10%) | 627 (62.26%) | 586 (58.19%) | **0.046** |
| Statins | 1863 (61.67%) | 619 (61.47%) | 640 (63.56%) | 604 (59.98%) | 0.253 |
| Fenofibrate | 4 (0.13%) | 1 (0.10%) | 1 (0.10%) | 2 (0.20%) | 0.999 |
| Other lipid-lowering drugs | 232 (7.68%) | 82 (8.14%) | 67 (6.65%) | 83 (8.24%) | 0.325 |
| ACEI/ARB | 1354 (44.82%) | 432 (42.90%) | 470 (46.67%) | 452 (44.89%) | 0.234 |
| ARNI | 30 (0.99%) | 10 (0.99%) | 6 (0.60%) | 14 (1.39%) | 0.199 |
| β‑blocker | 2163 (71.60%) | 687 (68.22%) | 735 (72.99%) | 741 (73.58%) | **0.014** |
| CCB | 722 (23.90%) | 225 (22.34%) | 252 (25.02%) | 245 (24.33%) | 0.342 |
| Mineralocorticoid antagonists | 1904 (63.03%) | 622 (61.77%) | 644 (63.95%) | 638 (63.36%) | 0.576 |
| Diuretics | 1454 (48.13%) | 461 (45.78%) | 485 (48.16%) | 508 (50.45%) | 0.111 |
| Nitrates | 1298 (42.97%) | 422 (41.91%) | 450 (44.69%) | 426 (42.30%) | 0.395 |
| Digoxin | 1046 (34.62%) | 332 (32.97%) | 369 (36.64%) | 345 (34.26%) | 0.213 |
| Insulin | 408 (13.51%) | 138 (13.70%) | 127 (12.61%) | 143 (14.20%) | 0.566 |
| SGLT2 inhibitors | 40 (1.32%) | 12 (1.19%) | 10 (0.99%) | 18 (1.79%) | 0.268 |
| Other oral antidiabetic agents | 668 (22.11%) | 223 (22.14%) | 220 (21.85%) | 225 (22.34%) | 0.964 |

*TyG index* triglyceride-glucose index, *PSM* propensity score matching, *BMI* body mass index, *SBP* systolic blood pressure, *DBP* diastolic blood pressure, *HR* heart rate, *LVEF* left ventricular ejection fraction*, NYHA* New York Heart Association, *AF* atrial fibrillation, *CKD* chronic kidney disease, *MI* myocardial infarction, *PAD* peripheral arterial disease, *COPD* chronic obstructive pulmonary disease, *MetS* metabolic syndrome, *PCI* percutaneous coronary intervention, CABG coronary artery bypass grafting, *WBC* white blood cell, *ALT* alanine aminotransferase, *AST* aspartate aminotransferase, *TBil* total bilirubin, *BUN* blood urea nitrogen, *eGFR* estimated glomerular filtration rate, *FBG* fasting blood glucose, *TG* triglyceride, *TC* total cholesterol, *LDL-C* low-density lipoprotein cholesterol, *HDL*-*C* high-density lipoprotein cholesterol, *LDH* lactic dehydrogenase, *cTnT* cardiac troponin T, *NT*-*proBNP* N-terminal pro-brain natriuretic peptide, *ACEI/ARB* angiotensin converting enzyme inhibitor/angiotensin receptor blocker, *ARNI* angiotensin receptor-neprilysin inhibitors, *CCB* calcium channel blockers, *SGLT2 inhibitors* sodium-glucose co-transporter-2 inhibitors, *CV death* cardiovascular death. *P* values < 0.05 are presented in bold

| **Table S2 HRs of primary outcomes according to TyG index tertiles after PSM analysis.** | | | | | | | | |
| --- | --- | --- | --- | --- | --- | --- | --- | --- |
| **Categories** | **Incidence/**  **1000 person-y** | **Univariate analysis** | | |  | **Multivariate analysis** | | |
|  |  | **HR (95% CI)** | ***P*-value** | ***P* for trend** |  | **HR (95% CI)** | ***P*-value** | ***P* for trend** |
| All-cause death |  |  |  |  |  |  |  |  |
| Continuousvariable per 1 unit |  | 1.40 (1.26-1.56) | **<0.001** |  |  | 1.80 (1.57-2.06) | **<0.001** |  |
| Tertile^a^ | 63.38 |  |  | **<0.001** |  |  |  | **<0.001** |
| T1 (n=1007) | 48.06 | Ref. |  |  |  | Ref. |  |  |
| T2 (n=1007) | 62.88 | 1.31 (1.10-1.55) | **0.002** |  |  | 1.44 (1.20-1.72) | **<0.001** |  |
| T3 (n=1007) | 80.63 | 1.67 (1.42-1.97) | **<0.001** |  |  | 2.10 (1.73-2.54) | **<0.001** |  |
| CV death |  |  |  |  |  |  |  |  |
| Continuousvariable per 1 unit |  | 1.47 (1.28-1.69) | **<0.001** |  |  | 1.77 (1.49-2.12) | **<0.001** |  |
| Tertile | 38.42 |  |  | **<0.001** |  |  |  | **<0.001** |
| T1 (n=1007) | 27.20 | Ref. |  |  |  | Ref. |  |  |
| T2 (n=1007) | 38.89 | 1.43 (1.14-1.78) | **0.002** |  |  | 1.53 (1.21-1.94) | **<0.001** |  |
| T3 (n=1007) | 50.11 | 1.84 (1.48-2.27) | .. **<0.001** |  |  | 2.14 (1.67-2.75) | **<0.001** |  |

*CI* confidence interval, *HR* hazard ratio, *TyG index* triglyceride-glucose index, *PSM* propensity score matching

Multivariate analysis: adjusted for age, gender, body mass index, heart rate, smoking status, white blood cell, hemoglobin, platelets, TBil, albumin, total cholesterol, LDL-C, HDL-C, sodium, previous cardiac valve surgery, antiplatelet agents and β-blocker.

^a^TyG index: T1 (< 8.40), T2 (8.40-8.93), T3 (≥ 8.93). *P* values < 0.05 are presented in bold

| **Table S3 HRs of primary outcomes according to TyG index tertiles in different metabolic status groups** | | | | | | | | | | | | |
| --- | --- | --- | --- | --- | --- | --- | --- | --- | --- | --- | --- | --- |
| **Subgroups** | **Events (%)** | **Unadjusted** |  |  |  | **Model 1** |  |  |  | **Model 2** |  |  |
|  |  | **HR (95% CI)** | ***P*-value** | ***P* for trend** |  | **HR (95% CI)** | ***P*-value** | ***P* for trend** |  | **HR (95% CI)** | ***P*-value** | ***P* for trend** |
| MetS Group |  |  |  |  |  |  |  |  |  |  |  |  |
| All-cause death |  |  |  |  |  |  |  |  |  |  |  |  |
| Continuous variable per 1 unit |  | 1.44 (1.31-1.57) | **<0.001** |  |  | 1.77 (1.60-1.97) | **<0.001** |  |  | 1.67 (1.50-1.87) | **<0.001** |  |
| Tertile^a^ | 1170 (35.8) |  |  | **<0.001** |  |  |  | **<0.001** |  |  |  | **<0.001** |
| T1 (n=1089) | 278 (25.5) | Ref. |  |  |  | Ref. |  |  |  | Ref. |  |  |
| T2 (n=1089) | 422 (38.8) | 1.67 (1.44-1.94) | **<0.001** |  |  | 1.88 (1.61-2.20) | **<0.001** |  |  | 1.90 (1.62-2.22) | **<0.001** |  |
| T3 (n=1089) | 470 (43.2) | 1.94 (1.67-2.25) | **<0.001** |  |  | 2.43 (2.07-2.86) | **<0.001** |  |  | 2.26 (1.91-2.66) | **<0.001** |  |
| CV death |  |  |  |  |  |  |  |  |  |  |  |  |
| Continuous variable per 1 unit |  | 1.44 (1.29-1.61) | **<0.001** |  |  | 1.74 (1.53-1.99) | **<0.001** |  |  | 1.66 (1.44-1.90) | **<0.001** |  |
| Tertile^a^ | 727 (22.3) |  |  | **<0.001** |  |  |  | **<0.001** |  |  |  | **<0.001** |
| T1 (n=1089) | 176 (16.2) | Ref. |  |  |  | Ref. |  |  |  | Ref. |  |  |
| T2 (n=1089) | 253 (23.2) | 1.57 (1.30-1.91) | **<0.001** |  |  | 1.81 (1.48-2.21) | **<0.001** |  |  | 1.81 (1.48-2.21) | **<0.001** |  |
| T3 (n=1089) | 298 (27.4) | 1.93 (1.60-2.33) | **<0.001** |  |  | 2.41 (1.96-2.95) | **<0.001** |  |  | 2.26 (1.84-2.79) | **<0.001** |  |
| Non-MetS Group |  |  |  |  |  |  |  |  |  |  |  |  |
| All-cause death |  |  |  |  |  |  |  |  |  |  |  |  |
| Continuous variable per 1 unit |  | 1.22 (1.07-1.39) | **..0.003** |  |  | 1.50 (1.30-1.73) | **<0.001** |  |  | 1.40 (1.19-1.64) | **<0.001** |  |
| Tertile^b^ | 988 (28.8) |  |  | **...0.009** |  |  |  | **<0.001** |  |  |  | **<0.001** |
| T1 (n=1143) | 316 (27.6) | Ref. |  |  |  | Ref. |  |  |  | Ref. |  |  |
| T2 (n=1143) | 312 (27.3) | 1.00 (0.85-1.17) | ...0.992 |  |  | 1.18 (1.00-1.39) | **...0.044** |  |  | 1.21 (1.02-1.42) | **...0.027** |  |
| T3 (n=1144) | 360 (31.5) | 1.23 (1.05-1.43) | **..0.008** |  |  | 1.52 (1.29-1.80) | **<0.001** |  |  | 1.41 (1.18-1.68) | **<0.001** |  |
| CV death |  |  |  |  |  |  |  |  |  |  |  |  |
| Continuous variable per 1 unit |  | 1.31 (1.11-1.55) | **..0.002** |  |  | 1.56 (1.29-1.87) | **<0.001** |  |  | 1.46 (1.19-1.80) | **<0.001** |  |
| Tertile^b^ | 578 (16.9) |  |  | **<0.001** |  |  |  | **<0.001** |  |  |  | **<0.001** |
| **Table S3 (continued)** | | | | | | | | | | | | |
| **Subgroups** | **Events (%)** | **Unadjusted** |  |  |  | **Model 1** |  |  |  | **Model 2** |  |  |
|  |  | **HR (95% CI)** | ***P*-value** | ***P* for trend** |  | **HR (95% CI)** | ***P*-value** | ***P* for trend** |  | **HR (95% CI)** | ***P*-value** | ***P* for trend** |
| T1 (n=1143) | 166 (14.5) | Ref. |  |  |  | Ref. |  |  |  | Ref. |  |  |
| T2 (n=1143) | 195 (17.1) | 1.19 (0.97-1.47) | ...0.096 |  |  | 1.40 (1.13-1.74) | **..0.002** |  |  | 1.47 (1.18-1.83) | **<0.001** |  |
| T3 (n=1144) | 217 (19.0) | 1.41 (1.15-1.73) | **<0.001** |  |  | 1.72 (1.38-2.15) | **<0.001** |  |  | 1.61 (1.27-2.04) | **<0.001** |  |

*CI* confidence interval, *HR* hazard ratio, *TyG index* triglyceride–glucose index, *MetS* metabolic syndrome

Model 1: adjusted for age, gender, body mass index, smoking status, drinking status, hemoglobin, ALT, AST, TBil, albumin, eGFR, total cholesterol, LDL-C, HDL-C, cTnT, sodium, LVEF, NT-proBNP and NYHA classification.

Model 2: adjusted for Model 1 + hypertension, diabetes, atrial fibrillation, previous MI, angina, stroke, COPD, previous heart surgery, antiplatelet agent, lipid-lowering drugs, ACEI/ARB, ARNI, β-blocker, mineralocorticoid antagonist, diuretics, digoxin and hypoglycemic therapy.

^a^TyG index: T1 (< 8.80), T2 (8.80-9.25), T3 (≥ 9.25); ^b^TyG index: T1 (< 8.14), T2 (8.14-8.56), T3 (≥ 8.56). *P* values < 0.05 are presented in bold

| **Table S4 HRs of primary outcomes according to TyG index tertiles in different heart failure phenotypes** | | | | | | | | | | | | |
| --- | --- | --- | --- | --- | --- | --- | --- | --- | --- | --- | --- | --- |
| **Subgroups** | **Events (%)** | **Unadjusted** |  |  |  | **Model 1** |  |  |  | **Model 2** |  |  |
|  |  | **HR (95% CI)** | ***P*-value** | ***P* for trend** |  | **HR (95% CI)** | ***P*-value** | ***P* for trend** |  | **HR (95% CI)** | ***P*-value** | ***P* for trend** |
| HFrEF Group |  |  |  |  |  |  |  |  |  |  |  |  |
| All-cause death |  |  |  |  |  |  |  |  |  |  |  |  |
| Continuous variable per 1 unit |  | 1.06 (0.95-1.19) | ...0.313 |  |  | 1.22 (1.06-1.39) | **..0.004** |  |  | 1.07 (0.92-1.25) | ...0.403 |  |
| Tertile^a^ | 776 (34.9) |  |  | ...0.262 |  |  |  | **..0.002** |  |  |  | ...0.170 |
| T1 (n=740) | 258 (34.9) | Ref. |  |  |  | Ref. |  |  |  | Ref. |  |  |
| T2 (n=740) | 252 (34.1) | 1.00 (0.84-1.19) | ...0.996 |  |  | 1.11 (0.92-1.33) | ...0.275 |  |  | 1.05 (0.87-1.27) | ...0.616 |  |
| T3 (n=741) | 266 (35.9) | 1.10 (0.93-1.31) | ...0.267 |  |  | 1.37 (1.13-1.67) | **..0.002** |  |  | 1.16 (0.94-1.45) | ...0.169 |  |
| CV death |  |  |  |  |  |  |  |  |  |  |  |  |
| Continuous variable per 1 unit |  | 1.07 (0.94-1.23) | ...0.299 |  |  | 1.22 (1.04-1.44) | **..0.014** |  |  | 1.08 (0.89-1.30) | ...0.430 |  |
| Tertile^a^ | 542 (24.4) |  |  | ...0.156 |  |  |  | **..0.003** |  |  |  | ...0.142 |
| T1 (n=740) | 173 (23.4) | Ref. |  |  |  | Ref. |  |  |  | Ref. |  |  |
| T2 (n=740) | 182 (24.6) | 1.08 (0.88-1.33) | ...0.464 |  |  | 1.18 (0.95-1.47) | ...0.131 |  |  | 1.10 (0.88-1.38) | ...0.400 |  |
| T3 (n=741) | 187 (25.2) | 1.16 (0.94-1.43) | ...0.156 |  |  | 1.44 (1.14-1.82) | **..0.003** |  |  | 1.22 (0.94-1.58) | ...0.142 |  |
| HFmrEF Group |  |  |  |  |  |  |  |  |  |  |  |  |
| All-cause death |  |  |  |  |  |  |  |  |  |  |  |  |
| Continuous variable per 1 unit |  | 1.35 (1.18-1.55) | **<0.001** |  |  | 1.74 (1.44-2.09) | **<0.001** |  |  | 1.52 (1.23-1.87) | **<0.001** |  |
| Tertile^b^ | 436 (30.4) |  |  | **<0.001** |  |  |  | **<0.001** |  |  |  | **..0.048** |
| T1 (n=478) | 122 (25.5) | Ref. |  |  |  | Ref. |  |  |  | Ref. |  |  |
| T2 (n=477) | 148 (31.0) | 1.22 (0.96-1.54) | ...0.112 |  |  | 1.36 (1.06-1.75) | **..0.017** |  |  | 1.27 (0.98-1.65) | ....0.071 |  |
| T3 (n=478) | 166 (34.7) | 1.49 (1.18-1.88) | **<0.001** |  |  | 1.64 (1.25-2.15) | **<0.001** |  |  | 1.36 (1.01-1.81) | **..0.041** |  |
| CV death |  |  |  |  |  |  |  |  |  |  |  |  |
| Continuous variable per 1 unit |  | 1.52 (1.27-1.82) | **<0.001** |  |  | 1.87 (1.46-2.39) | **<0.001** |  |  | 1.59 (1.20-2.10) | **0.001** |  |
| Tertile^b^ | 235 (16.4) |  |  | **<0.001** |  |  |  | **<0.001** |  |  |  | **..0.022** |
| **Table S4 (continued)** | | | | | | | | | | | | |
| **Subgroups** | **Events (%)** | **Unadjusted** |  |  |  | **Model 1** |  |  |  | **Model 2** |  |  |
|  |  | **HR (95% CI)** | ***P*-value** | ***P* for trend** |  | **HR (95% CI)** | ***P*-value** | ***P* for trend** |  | **HR (95% CI)** | ***P*-value** | ***P* for trend** |
| T1 (n=478) | 59 (12.3) | Ref. |  |  |  | Ref. |  |  |  | Ref. |  |  |
| T2 (n=477) | 76 (15.9) | 1.29 (0.92-1.82) | ...0.139 |  |  | 1.40 (0.98-1.99) | ..0.065 |  |  | 1.38 (0.95-2.00) | ...0.091 |  |
| T3 (n=478) | 100 (20.9) | 1.85 (1.34-2.55) | **<0.001** |  |  | 1.92 (1.32-2.79) | **<0.001** |  |  | 1.62 (1.08-2.43) | **. .0.019** |  |
| HFpEF Group |  |  |  |  |  |  |  |  |  |  |  |  |
| All-cause death |  |  |  |  |  |  |  |  |  |  |  |  |
| Continuous variable per 1 unit |  | 1.76 (1.61-1.93) | **<0.001** |  |  | 1.98 (1.77-2.22) | **<0.001** |  |  | 1.94 (1.71-2.21) | **<0.001** |  |
| Tertile^c^ | 946 (31.1) |  |  | **<0.001** |  |  |  | **<0.001** |  |  |  | **<0.001** |
| T1 (n=1014) | 182 (17.9) | Ref. |  |  |  | Ref. |  |  |  | Ref. |  |  |
| T2 (n=1014) | 306 (30.2) | 1.78 (1.48-2.14) | **<0.001** |  |  | 1.83 (1.51-2.22) | **<0.001** |  |  | 1.68 (1.38-2.05) | **<0.001** |  |
| T3 (n=1015) | 458 (45.1) | 3.07 (2.59-3.65) | **<0.001** |  |  | 3.36 (2.74-4.11) | **<0.001** |  |  | 3.07 (2.47-3.81) | **<0.001** |  |
| CV death |  |  |  |  |  |  |  |  |  |  |  |  |
| Continuous variable per 1 unit |  | 1.96 (1.74-2.20) | **<0.001** |  |  | 2.14 (1.84-2.49) | **<0.001** |  |  | 2.12 (1.79-2.51) | **<0.001** |  |
| Tertile^c^ | 528 (17.4) |  |  | **<0.001** |  |  |  | **<0.001** |  |  |  | **<0.001** |
| T1 (n=1014) | 84 (8.3) | Ref. |  |  |  | Ref. |  |  |  | Ref. |  |  |
| T2 (n=1014) | 165 (16.3) | 2.07 (1.59-2.69) | **<0.001** |  |  | 2.11 (1.60-2.78) | **<0.001** |  |  | 1.95 (1.47-2.60) | **<0.001** |  |
| T3 (n=1015) | 279 (27.5) | 4.03 (3.16-5.15) | **<0.001** |  |  | 4.25 (3.21-5.65) | **<0.001** |  |  | 3.93 (2.91-5.32) | **<0.001** |  |

*CI* confidence interval, *HR* hazard ratio, *TyG index* triglyceride–glucose index, *HFrEF* heart failure with reduced ejection fraction, *HFmrEF* heart failure with mildly reduced ejection fraction, *HFpEF* heart failure with preserved ejection fraction. *P* values < 0.05 are presented in bold

Model 1: adjusted for age, gender, body mass index, smoking status, drinking status, hemoglobin, ALT, AST, TBil, albumin, eGFR, total cholesterol, LDL-C, HDL-C, cTnT, sodium, LVEF, NT-proBNP and NYHA classification.

Model 2: adjusted for Model 1 + hypertension, diabetes, atrial fibrillation, previous MI, angina, stroke, COPD, previous heart surgery, antiplatelet agent, lipid-lowering drugs, ACEI/ARB, ARNI, β-blocker, mineralocorticoid antagonist, diuretics, digoxin and hypoglycemic therapy.

^a^TyG index: T1 (< 8.33), T2 (8.33-8.83), T3 (≥ 8.83); ^b^TyG index: T1 (< 8.43), T2 (8.43-8.94), T3 (≥ 8.94); ^c^TyG index: T1 (< 8.44), T2 (8.44-8.98), T3 (≥ 8.98)

| **Table S5 HRs of primary outcomes according to TyG index tertiles among other different subgroups** | | | | | | | | | |
| --- | --- | --- | --- | --- | --- | --- | --- | --- | --- |
| **Subgroups** | **Model 1** | | |  | **Model 2** | | |  | ***P* for**  **interaction** |
|  | **HR (95% CI)** | ***P*-value** | ***P* for trend** |  | **HR (95% CI)** | ***P*-value** | ***P* for trend** |  |  |
| Diabetes group |  |  |  |  |  |  |  |  |  |
| All-cause death |  |  | **<0.001** |  |  |  | **<0.001** |  | 0.686^a^ |
| T1 (n=996) | Ref. |  |  |  | Ref. |  |  |  |  |
| T2 (n=995) | 1.32 (1.14-1.52) | **<0.001** |  |  | 1.45 (1.25-1.68) | **<0.001** |  |  |  |
| T3 (n=996) | 1.68 (1.46-1.93) | **<0.001** |  |  | 1.95 (1.67-2.29) | **<0.001** |  |  |  |
| CV death |  |  | **<0.001** |  |  |  | **<0.001** |  | 0.484^a^ |
| T1 (n=996) | Ref. |  |  |  | Ref. |  |  |  |  |
| T2 (n=995) | 1.37 (1.14-1.65) | .. **<0.001** |  |  | 1.51 (1.24-1.83) | . **<0.001** |  |  |  |
| T3 (n=996) | 1.82 (1.52-2.18) | .. **<0.001** |  |  | 2.13 (1.74-2.60) | . **<0.001** |  |  |  |
| Non-diabetes group |  |  |  |  |  |  |  |  |  |
| All-cause death |  |  | **0.005** |  |  |  | **<0.001** |  |  |
| T1 (n=1237) | Ref. |  |  |  | Ref. |  |  |  |  |
| T2 (n=1236) | 1.08 (0.92-1.26) | 0.376 |  |  | 1.23 (1.04-1.45) | **0.018** |  |  |  |
| T3 (n=1237) | 1.25 (1.07-1.47) | **0.005** |  |  | 1.54 (1.27-1.86) | **<0.001** |  |  |  |
| CV death |  |  | **0.004** |  |  |  | **<0.001** |  |  |
| T1 (n=1237) | Ref. |  |  |  | Ref. |  |  |  |  |
| T2 (n=1236) | 1.23 (1.00-1.52) | **0.047** |  |  | 1.43 (1.15-1.79) | **0.001** |  |  |  |
| T3 (n=1237) | 1.36 (1.10-1.67) | **0.004** |  |  | 1.66 (1.29-2.13) | **. <0.001** |  |  |  |
| HTN group |  |  |  |  |  |  |  |  |  |
| All-cause death |  |  | **<0.001** |  |  |  | **<0.001** |  | 0.041^b^ |
| T1 (n=1381) | Ref. |  |  |  | Ref. |  |  |  |  |
| T2 (n=1380) | 1.38 (1.20-1.59) | **<0.001** |  |  | 1.35 (1.16-1.57) | **<0.001** |  |  |  |
| T3 (n=1381) | 2.14 (1.87-2.44) | **<0.001** |  |  | 2.02 (1.71-2.38) | **<0.001** |  |  |  |
| CV death |  |  | **<0.001** |  |  |  | **<0.001** |  | 0.055^b^ |
| T1 (n=1381) | Ref. |  |  |  | Ref. |  |  |  |  |
| T2 (n=1380) | 1.42 (1.18-1.71) | **<0.001** |  |  | 1.33 (1.09-1.63) | **0.005** |  |  |  |
| T3 (n=1381) | 2.36 (1.98-2.81) | **<0.001** |  |  | 2.05 (1.66-2.54) | **<0.001** |  |  |  |
| Non-HTN group |  |  |  |  |  |  |  |  |  |
| All-cause death |  |  | **<0.001** |  |  |  | **<0.001** |  |  |
| T1 (n=852) | Ref. |  |  |  | Ref. |  |  |  |  |
| T2 (n=851) | 1.21 (1.01-1.45) | **0.034** |  |  | 1.31 (1.08-1.58) | **0.006** |  |  |  |
| T3 (n=852) | 1.51 (1.26-1.80) | **<0.001** |  |  | 1.47 (1.18-1.83) | **<0.001** |  |  |  |
| CV death |  |  | **<0.001** |  |  |  | **<0.001** |  |  |
| T1 (n=852) | Ref. |  |  |  | Ref. |  |  |  |  |
| T2 (n=851) | 1.32 (1.05-1.66) | **0.020** |  |  | 1.45 (1.14-1.86) | **0.003** |  |  |  |
| T3 (n=852) | 1.64 (1.31-2.06) | **<0.001** |  |  | 1.71 (1.29-2.27) | **<0.001** |  |  |  |
| Obesity group |  |  |  |  |  |  |  |  |  |
| All-cause death |  |  | **<0.001** |  |  |  | **<0.001** |  | 0.678^c^ |
| T1 (n=539) | Ref. |  |  |  | Ref. |  |  |  |  |
| T2 (n=539) | 2.02 (1.59-2.56) | **<0.001** |  |  | 2.47 (1.90-3.21) | **<0.001** |  |  |  |
| **Table S5 (continued)** | | | | | | | | | |
| **Subgroups** | **Model 1** | | **Model 2** | | | | |  | ***P* for interaction** |
|  | **HR (95% CI)** | ***P*-value** | ***P* for trend** |  | **HR (95% CI)** | ***P*-value** | ***P* for trend** |  |  |
| T3 (n=539) | 2.30 (1.82-2.91) | **<0.001** |  |  | 3.13 (2.36-4.16) | **<0.001** |  |  |  |
| CV death |  |  | **<0.001** |  |  |  | **<0.001** |  | 0.924^c^ |
| T1 (n=539) | Ref. |  |  |  | Ref. |  |  |  |  |
| T2 (n=539) | 2.10 (1.55-2.85) | **<0.001** |  |  | 2.47 (1.77-3.46) | **<0.001** |  |  |  |
| T3 (n=539) | 2.33 (1.72-3.15) | **<0.001** |  |  | 2.96 (2.06-4.26) | **<0.001** |  |  |  |
| Non-obesity group |  |  |  |  |  |  |  |  |  |
| All-cause death |  |  | **<0.001** |  |  |  | **<0.001** |  |  |
| T1 (n=1693) | Ref. |  |  |  | Ref. |  |  |  |  |
| T2 (n=1693) | 1.19 (1.05-1.35) | **0.007** |  |  | 1.20 (1.05-1.37) | **0.009** |  |  |  |
| T3 (n=1694) | 1.74 (1.54-1.96) | **<0.001** |  |  | 1.69 (1.46-1.97) | **<0.001** |  |  |  |
| CV death |  |  | **<0.001** |  |  |  | **<0.001** |  |  |
| T1 (n=1693) | Ref. |  |  |  | Ref. |  |  |  |  |
| T2 (n=1693) | 1.28 (1.08-1.51) | **0.004** |  |  | 1.27 (1.06-1.52) | **0.008** |  |  |  |
| T3 (n=1694) | 1.93 (1.65-2.26) | **<0.001** |  |  | 1.91 (1.57-2.33) | **<0.001** |  |  |  |
| Dyslipidemia group |  |  |  |  |  |  |  |  |  |
| All-cause death |  |  | **<0.001** |  |  |  | **<0.001** |  | 0.977^d^ |
| T1 (n=1395) | Ref. |  |  |  | Ref. |  |  |  |  |
| T2 (n=1395) | 1.31 (1.13-1.50) | **<0.001** |  |  | 1.45 (1.25-1.69) | **<0.001** |  |  |  |
| T3 (n=1396) | 1.78 (1.56-2.04) | **<0.001** |  |  | 1.95 (1.67-2.29) | **<0.001** |  |  |  |
| CV death |  |  | **<0.001** |  |  |  | **<0.001** |  | 0.779^d^ |
| T1 (n=1395) | Ref. |  |  |  | Ref. |  |  |  |  |
| T2 (n=1395) | 1.30 (1.08-1.55) | **0.004** |  |  | 1.45 (1.20-1.75) | **<0.001** |  |  |  |
| T3 (n=1396) | 1.80 (1.52-2.13) | **<0.001** |  |  | 1.97 (1.61-2.40) | **<0.001** |  |  |  |
| Non-dyslipidemia group |  |  |  |  |  |  |  |  |  |
| All-cause death |  |  | **<0.001** |  |  |  | **<0.001** |  |  |
| T1 (n=837) | Ref. |  |  |  | Ref. |  |  |  |  |
| T2 (n=837) | 1.20 (1.00-1.44) | **0.050** |  |  | 1.31 (1.08-1.58) | **0.007** |  |  |  |
| T3 (n=837) | 1.78 (1.50-2.11) | **<0.001** |  |  | 1.76 (1.43-2.17) | **<0.001** |  |  |  |
| CV death |  |  | **<0.001** |  |  |  | **<0.001** |  |  |
| T1 (n=837) | Ref. |  |  |  | Ref. |  |  |  |  |
| T2 (n=837) | 1.34 (1.05-1.70) | **0.020** |  |  | 1.41 (1.09-1.83) | **0.009** |  |  |  |
| T3 (n=837) | 1.81 (1.44-2.28) | **<0.001** |  |  | 1.78 (1.35-2.36) | **<0.001** |  |  |  |
| Ischemic etiology |  |  |  |  |  |  |  |  |  |
| All-cause death |  |  | **<0.001** |  |  |  | **<0.001** |  | 0.030^e^ |
| T1 (n=1059) | Ref. |  |  |  | Ref. |  |  |  |  |
| T2 (n=1059) | 1.27 (1.09-1.48) | **0.002** |  |  | 1.26 (1.07-1.49) | **0.006** |  |  |  |
| T3 (n=1059) | 1.82 (1.58-2.11) | **<0.001** |  |  | 1.68 (1.40-2.03) | **<0.001** |  |  |  |
| CV death |  |  | **<0.001** |  |  |  | **<0.001** |  | 0.268^e^ |
| T1 (n=1059) | Ref. |  |  |  | Ref. |  |  |  |  |
| **Table S5 (continued)** | | | | | | | | | |
| **Subgroups** | **Model 1** | | |  | **Model 2** | | |  | ***P* for interaction** |
|  | **HR (95% CI)** | ***P*-value** | ***P* for trend** |  | **HR (95% CI)** | ***P*-value** | ***P* for trend** |  |  |
| T2 (n=1059) | 1.37 (1.12-1.67) | **0.002** |  |  | 1.33 (1.07-1.65) | **0.010** |  |  |  |
| T3 (n=1059) | 2.03 (1.69-2.45) | **<0.001** |  |  | 1.85 (1.46-2.35) | **<0.001** |  |  |  |
| Non-ischemic etiology |  |  |  |  |  |  |  |  |  |
| All-cause death |  |  | **<0.001** |  |  |  | **<0.001** |  |  |
| T1 (n=1173) | Ref. |  |  |  | Ref. |  |  |  |  |
| T2 (n=1173) | 1.41 (1.19-1.66) | **<0.001** |  |  | 1.46 (1.23-1.74) | **<0.001** |  |  |  |
| T3 (n=1174) | 2.06 (1.76-2.40) | **<0.001** |  |  | 2.26 (1.87-2.73) | **<0.001** |  |  |  |
| CV death |  |  | **<0.001** |  |  |  | **<0.001** |  |  |
| T1 (n=1173) | Ref. |  |  |  | Ref. |  |  |  |  |
| T2 (n=1173) | 1.64 (1.33-2.03) | **<0.001** |  |  | 1.72 (1.37-2.17) | **<0.001** |  |  |  |
| T3 (n=1174) | 2.18 (1.77-2.68) | **<0.001** |  |  | 2.47 (1.92-3.18) | **<0.001** |  |  |  |

*CI* confidence interval, *HR* hazard ratio, *TyG index* triglyceride–glucose index, *HTN* hypertension

Model 1: adjusted for age and gender.

Model 2: adjusted for Model 1 + body mass index, smoking status, drinking status, hemoglobin, ALT, AST, TBil, albumin, eGFR, total cholesterol, LDL-C, HDL-C, cTnT, sodium, LVEF, NT-proBNP, NYHA classification, hypertension, diabetes, atrial fibrillation, previous MI, angina, stroke, COPD, previous heart surgery, antiplatelet agents, lipid-lowering drugs, ACEI/ARB, ARNI, β-blocker, mineralocorticoid antagonist, diuretics, digoxin and hypoglycemic therapy.

According to the diagnostic criteria of metabolic syndrome in this study, the obesity was defined by BMI ≥ 28 kg/m^2^ and the dyslipidemia was defined by fasting TG ≥ 1.7 mmol/L or fasting HDL-C < 1.04 mmol/L. Ischemic etiology included the previous myocardial infarction, angina, the history of PCI or CABG surgery.

^a^*P* for interaction between the TyG index and the diabetes with all-cause death and CV death as endpoint, respectively.

^b^*P* for interaction between the TyG index and the hypertension with all-cause death and CV death as endpoint, respectively.

^c^*P* for interaction between the TyG index and the obesity with all-cause death and CV death as endpoint, respectively.

^d^*P* for interaction between the TyG index and the dyslipidemia with all-cause death and CV death as endpoint, respectively.

^e^*P* for interaction between the TyG index and the ischemic etiology with all-cause death and CV death as endpoint, respectively.
